# Nitrobenzoates and Nitrothiobenzoates with Activity against *M. tuberculosis*

**DOI:** 10.3390/microorganisms11040969

**Published:** 2023-04-08

**Authors:** João P. Pais, Olha Antoniuk, Raquel Freire, David Pires, Emília Valente, Elsa Anes, Luis Constantino

**Affiliations:** 1 Research Institute for Medicines (iMed.UL), Av. Prof. Gama Pinto, 1649-003 Lisboa, Portugal; 2 Faculty of Pharmacy, Universidade de Lisboa, Av. Prof. Gama Pinto, 1649-003 Lisboa, Portugal; 3 Center for Interdisciplinary Research in Health, Católica Medical School, Universidade Católica Portuguesa, Estrada Octávio Pato, 2635-631 Rio de Mouro, Portugal

**Keywords:** benzoates, nitrobenzoates, tuberculosis, prodrugs, esterases, mycobacteria

## Abstract

Esters of weak acids have shown improved antimycobacterial activity over the corresponding free acids and nitro benzoates in particular have previously shown to have a very intriguing activity. To expand the potential of nitro-derivatives of benzoic acid as antimycobacterial drugs and explore the effects of various structural features on the activity of these compounds, we have obtained a library of 64 derivatives containing esters and thioesters of benzoates and studied their activity against *M. tuberculosis*, the stability of the compounds, their activation by mycobacterial enzymes and the potential cytotoxicity against human monocytic THP-1 cell line. Our results showed that the most active compounds are those with an aromatic nitro substitution, with the 3,5-dinitro esters series being the most active. Also, the greater antitubercular activity for the nitro derivatives was shown to be unrelated to their pKa values or hydrolysis rates. Given the conventional relationship between nitro-containing substances and toxicity, one might anticipate that the great antimicrobial activity of nitro compounds would be associated with high toxicity; yet, we have not found such a relationship. The nitrobenzoate scaffold, particularly the 3,5-dinitrobenzoate scaffold, merits further investigation, because it has the potential to generate future antimycobacterial agents with improved activity.

## 1. Introduction

Tuberculosis (TB) has been a menace to mankind for ages [1]. Evidence of *Mycobacterium tuberculosis* (Mtb) infection was found in ancient skeletal and mummified material, including Egyptian mummies from 3000 to 2400 BC [2]. The discovery of antibiotics helped to control the infection to some extent, but it remains one of the deadliest infectious diseases, primarily affecting those who suffer from malnutrition, comorbidities such as diabetes, or immunosuppressive conditions. The situation is getting worse due to the ongoing spread of multidrug-resistant Mtb strains and the fact that TB services have been disproportionately affected by the COVID pandemic [3]. According to the World Health Organization (WHO), case notifications have plunged due to pandemic-related disruptions, and, due to a lack of access to care, TB mortality has increased for the first time in more than ten years [4].

The path to recovery requires a multifaceted approach and new therapeutic options. Pathogenic mycobacteria are known to be sensitive to acid media, as was demonstrated previously by our group [5]. Furthermore, mutant bacteria that were unable to stop macrophage phagosome maturation were eliminated in late endosomal compartments [6,7], where the acidic pH (pH = 5.8) hinders bacterial multiplication [6,8]. When compared to other bacterial species, Mtb is particularly more vulnerable to a variety of weak acids, and this acid sensitivity can be exacerbated by several stressors [9,10].

Our team has recently investigated the antimycobacterial potential of benzoic acid and its derivatives, particularly against Mtb, attempting to develop alkyl esters as prodrugs capable of leveraging the weak acids’ antitubercular activity. It was hypothesized that the pKa of weak acids could be related to the activity of the compounds [9,10], and esters could act as prodrugs of the weak acid facilitating the entrance of the compounds into mycobacterial cells, where they could be hydrolysed and, therefore, activated by mycobacterial esterases. We have previously shown that mycobacteria can swiftly hydrolyse a variety of organic acid esters [11,12] and that esters possess greater in vitro activity than the corresponding free organic acids [13,14]. Altogether, this indicates that they are suitable prodrugs for the compounds, facilitating the entry of the molecules and liberating the free acid inside the cells.

When testing a series of acids and esters with different pKa, we could not find a relationship between pKa and activity; however, we did find that esters of benzoic acid containing 4-nitro or 3,5-dinitro groups in the aromatic ring demonstrate greater activity than esters of derivatives containing other substituents and similar pKa [15].

While it is known that weak organic acids show good broad-spectrum antimicrobial activities [16] and Mtb has an increased sensitivity to an acid environment, aromatic nitro derivatives seem to have a particular capacity to inhibit Mtb strains. In fact, it was demonstrated that 4-nitrobenzoic acid is capable of inhibiting pathogen growth in vitro and has been used to identify species of the *Mycobacterium tuberculosis* complex (MtbC). Another example of Mtb sensitivity to nitro groups is the emergence of covalent inhibitors of the enzyme DprE1, an essential target, where the nitro group is crucial to the mode of action [17]. The sensitivity to nitro-containing compounds of the MtbC bacteria could be attributed to their deficient metabolism when compared to other mycobacteria than MtbC which express higher concentrations of nitroreductase enzymes [18].

In the present work, we obtained a library of nitro derivatives of benzoic acid with different ester moieties ranging from short (with 3 carbon atoms) to long (with 16 carbon atoms) alkoxy chains. Their stability in buffer and human serum, as well as their facility of hydrolysis in mycobacterial homogenate, was assessed. Our aim was to explore the potential of nitro-derivatives of benzoic acid as antimycobacterial agents, studying the impact of multiple structural features on the activity and toxicity of these compounds. In order to further test these relationships, a smaller library of thioesters was synthesized, and compared with the esters concerning activity, stability, activation, and toxicity.

## 2. Materials and Methods

Materials. The balanced salt solution, phosphate-buffered saline (PBS), Dulbecco’s modified Eagle’s medium (DMEM), and L-glutamine were purchased from Invitrogen (Carlsbad, CA, USA). Sodium dodecyl sulphate (SDS), Triton X-100, benzoic acid, 4-chlorobenzoic acid, 3,5-dichlorobenzoic acid, 4-nitrobenzoic acid, 3,5-dinitrobenzoic acid, 4-(trifluoromethyl)benzoic acid, 3,5-bis(trifluoromethyl)benzoic acid, 2,3,4,5,6-pentafluorobenzoic acid, n-butanol, n-pentanol, n-hexanol, n-heptanol, n-octanol, n-nonanol, nonan-2-ol, n-decanol, n-undecanol, n-dodecanol, n-tridecanol, n-tetradecanol, n-hexadecanol, butanethiol, octanethiol, dodecanethiol, and trypan blue were purchased from Sigma-Aldrich (St. Louis, MO, USA). Middlebrook 7H10 agar was purchased from BD Difco (Franklin Lakes, NJ, USA). The materials and equipment used for antibiotic susceptibility tests of *M. tuberculosis* with the Bactec MGIT 960 PZA kit system were purchased from Becton Dickinson (Franklin Lakes, NJ, USA) and prepared according to the recommendations of the manufacturer. Microwell tissue culture plates were purchased from Nunc - Thermo Fisher Scientific (Waltham, MA, USA). Esters were synthesized according to the procedures described in this paper. Compounds were prepared in stock solutions of 40 mg/mL in dimethyl sulfoxide (DMSO—AppliChem Panreac, Barcelona, Spain). Isoniazid is a first-line antibiotic against tuberculosis and was used as a positive control for *M. tuberculosis* killing. Bacteria broth culture medium Middlebrook 7H9 and solid culture medium Middlebrook 7H10 were purchased from Difco (Franklin Lakes, NJ, USA) and were supplemented with OADC (oleic acid, albumin, dextrose, catalase) and tyloxapol. Isoniazid, OADC and tyloxapol were purchased from Sigma-Aldrich (St. Louis, MO, USA).

Bacterial strains and cell lines. *Mycobacterium tuberculosis* H37Rv (ATCC 27294) were cultivated in Middlebrook 7H9 medium supplemented with OADC and 0.05% tyloxapol and incubated at 37 °C until exponential growth phase was achieved.

Ester synthesis. Acyl chloride synthesis: A solution of the chosen benzoic acid derivative in thionyl chloride (3 mL per mmol of acid) was refluxed for 5 h, leading to the formation of the desired acyl chloride. The excess thionyl chloride was removed by low-pressure evaporation. The product was used without further purification.

Ester synthesis (method A): A solution of the appropriate acyl chloride (1.2 mmol per mmol of alcohol) in dichloromethane (DCM) was added dropwise to a solution of corresponding alcohol and triethylamine (TEA) in DCM at 0 °C. When the reaction was complete (as assessed by thin-layer chromatography (TLC) using hexane:ethyl acetate, 9:1 as eluent) the solvent was evaporated and the crude purified by column chromatography (silica gel 60) using hexane:ethyl acetate, 9.5:0.5 to 9:1 as eluent. This method was applied for the synthesis of compounds **5**–**20**, **26**–**33**, **39**–**44**, **47**.

Ester synthesis (method B): To a solution of the appropriate benzoic acid derivative (1.2 mmol per mmol of alcohol) in THF (10 mL per mmol of acid) carbonyldiimidazole (1 equiv.) was added. The reaction mixture was stirred at room temperature for 3.5 h. Then, the desired alcohol was added and the reaction mixture was refluxed until the completion of the reaction as assessed by TLC (hexane:ethyl acetate, 9:1). The solvent was evaporated, and the product purified by column chromatography (silica gel 60) using hexane:ethyl acetate, 10:0 to 9.6:0.4 as eluent. This method was applied for the synthesis of compounds **35**, **36**, **38**, **45**, and **46**.

Ester synthesis (method C/D): The benzoic acid derivative was dissolved in the desired alcohol (25 equiv.) and placed under stirring. Then, thionyl chloride (1.5 equiv. in method C) or sulfuric acid (0.5 equiv. in method D) were added dropwise, and the reaction was heated for 5 h at 50 °C (for method C) or 24 h at 120 °C (for method D) to form the respective esters. The reaction was followed by TLC (toluene as eluent) until the reaction was complete. Afterwards, water was added, and the ester was extracted with DCM. The organic solution was then washed with saturated sodium bicarbonate solution, dried with anhydrous sodium sulfate (Na_2_SO_4_), and evaporated. The ester was then purified by column chromatography (silica gel 60) using toluene as eluent. Method C was applied for the synthesis of compounds **1**–**4**; Method D was applied for compounds **22**, **23**, **25**, and **48**–**52**.

Thioester synthesis: A solution of the desired thiol (3 equiv.) and triethylamine (3,3 equiv.) in DCM was stirred at room temperature for 15 min. After, a solution of the appropriate acyl chloride in DCM was slowly added, dropwise. The reaction was followed by TLC until completion. Then, the solvent was evaporated, and the crude was purified by column chromatography (eluent: 9.5:0.5, hexane:ethyl acetate), yielding the desired thioester. This method was applied for the synthesis of compounds **53**–**64**.

Structural characterization of all compounds synthesized can be found in the Supplementary Materials.

HPLC analysis: Two HPLC systems were used. The buffer and plasma stability studies were performed in an HPLC system with a photodiode detector L-3000 Photo Diode Array Detector, Merck-Hitachi L-6000 pump, Merck-Hitachi D-2500 integrator, and Merck RP-8 column. Activation studies were performed in an HPLC system with a UV detector Merck-Hitachi UV-L7400, Merck Hitachi L-7100 pump, an autosampler Merck-Hitachi AS 2000, and a Merck-Hitachi D-2500 integrator and Merck RP-8 column. The eluant was a mixture of acetonitrile (60%/70%) and aqueous phosphate buffer with 5% of KH_2_PO_4_/H_3_PO_4_ 0.025 M (40%/30%). The flow rate was always 1 mL min^−1^ and the wavelength was set to 230 nm. All quantifications were evaluated using calibration curves from stock solutions.

Mycobacterial homogenate preparation: A crude whole mycobacterial homogenate was prepared according to reference [19]. A culture of exponentially growing *M. smegmatis* ATCC607 variant mc^2^ 155 with an O.D. 600 nm of 0.8–1.0 was harvested by centrifugation at T = 4 °C for 10 min, washed, and re-suspended in pH = 7.4 phosphate buffer saline PBS (25 mL for each 750 mL of the initial growing broth). The bacterial homogenate was prepared using an ultrasound probe with a sequence of five cycles of 2 mins each. The homogenate was afterwards divided into 1 mL portions and kept at −80 °C until use. Total protein concentration was 1.4 mg mL^−1^.

Conditions of incubations and preparation of samples: The initial substrate concentration was 5 × 10^−4^ M in all stability assays. All incubations were carried out at pH 7.4 and 37 °C under agitation using the phosphate buffer described above as diluting agent. The levels of dilution of the mycobacterial homogenate (20%) and human plasma (80%) in the incubates were chosen following preliminary assays to ensure pseudo-first-order kinetics in the hydrolysis of benzoates. Acetonitrile (2%) was used in all studies to ensure adequate solubilization of the substrates. The benzoates were added from 2.5 × 10^−2^ M acetonitrile stock solutions. After incubation, aliquots of 50 μL were taken into vials containing 450 μL of a 1:1 solution of 1% zinc sulphate and acetonitrile, mixed in a vortex and centrifuged for 10 min at 15,000 rpm. The supernatant was then injected into the HPLC and analysed for quantification of benzoic acid and remaining benzoate. All quantifications were performed using calibration curves.

Buffer stability. 1176 µL pH 7.4 phosphate buffer or pH 5.9 phosphate buffer, 18 µL ACN, and 6 µL of a 10^−1^ M stock solution of the prodrug in ACN were mixed in a 2 mL vial. The solution was incubated at 37 °C, and 50 µL aliquots were removed, mixed with 450 µL of ACN: H_2_O 1:1. The solution was analysed by HPLC—the remaining substrate and the correspondent acid were both measured.

Plasma stability. Pooled human plasma (960 µL), pH 7.4 phosphate-buffered saline (216 µL), 18 µL ACN and 6 µL of a 10^−1^ M stock solution of the prodrug in ACN were mixed in a 2 mL vial. The suspensions were incubated at 37 °C, and 50 µL aliquots were removed, mixed with 450 µL of ACN: ZnSO_4_ 1% 1:1, and centrifuged. The supernatant was removed and analysed by HPLC. The remaining substrate and the corresponding acid were measured.

Determination of the Minimum Inhibitory Concentration (MIC) and Minimum Bactericidal Concentrations (MBC): The MICs were determined by the broth microdilution method in 96-well plates. Briefly, *M. tuberculosis* bacterial cultures in the exponential growth phase were collected by centrifugation, washed in PBS, and re-suspended in fresh culture medium. Clumps of bacteria were removed by ultrasonic treatment of the bacteria suspension in an ultrasonic water bath for 5 min followed by a low-speed centrifugation (500× *g*) for 2 min. The single-cell suspension was verified by microscopy. The microplates containing a bacterial suspension corresponding to approximately 10^5^ colony-forming units per mL were incubated with the selected concentrations of the compounds. Every other day, the optical density of the wells was measured in a Tecan M200 spectrophotometer, following 30 s of orbital agitation. These values were used to produce the growth curves. On the 10th day of incubation, the MIC was determined, corresponding to the concentration with no visible turbidity. Optical density measures were taken until the 15th day of incubation, following which the bacterial samples were recovered from the MIC test microplates and plated in 7H10 + OADC solid medium. The MBC was determined following 3 weeks of incubation, corresponding to the concentration of compound that produced no colonies on the solid medium. Bacteria treated with DMSO solvent at the same proportions as present during the compound tests were used as a control. Isoniazid was used as a positive control for bacteria killing and assay validation following EUCAST guidelines (MIC = [0.03, 0.12]).

Determination of macrophage viability after treatment with compounds: THP-1 Human monocytic cell line THP-1 (ATCC TIB202) was used to determine the effect of the compounds on cell viability. The cells were grown in RPMI 1640 (Gibco) supplemented with 10% fetal bovine serum (FBS; Gibco), 10 mM HEPES (Gibco), 1 mM sodium pyruvate, and maintained at 37 °C with 5% CO_2_. Differentiation of THP-1 monocytes into macrophages was induced for 48 h with 20 nM phorbol 12-myristate 13-acetate (PMA) following a 24 h resting period without PMA. Differentiated macrophages in 96-well plates, 5 × 10^4^ cells per well, were treated with the compounds. After three days of treatment, cell viability was determined using PrestoBlue (Invitrogen, Carlsbad, CA, USA) following the manufacturer’s indications. Briefly, the cells were washed with PBS and incubated with PrestoBlue 10% (*v*/*v*) in cell culture medium. After 4 h of incubation, the fluorescence of each well was measured in a Tecan M200 spectrophotometer (Em: 560 nm/Ex: 590 nm). Viability was calculated relatively to non-treated cells. Cells treated with DMSO solvent at the same proportions were used as a control. Puromycin was used as a positive control for cell death. 

## 3. Results

### 3.1. Activity against M. tuberculosis

#### 3.1.1. Esters

Table 1 presents the structures of the esters tested in the present study. Compounds differ in the aromatic substituents and in the ester alkoxy group. For each aromatic substituent, a series of esters with different lipophilicities was prepared. For comparison purposes, several esters made from acids of similar pKa but not containing nitro substituents were included. Simple aromatic unsubstituted benzoates were also included in the study. Table 1 also displays activity expressed as MIC and MBC values against the Mtb H37Rv strain.

An analysis of the data in Table 1 reveals that the substances having nitro substitution in the aromatic ring are those that are most active, with the 3,5-dinitro esters series being the most active overall. All nitro-substituted series presented activities higher than the series made from acids of comparable pKa. For instance, 4-nitro derivatives (pKa 3.4) liberate a free acid with pKa of 3.4 but have activities greater than the other derivatives that liberate acid with similar pKa like 3,5-dichloro (pKa 3,6), 4-trifluoromethyl (pKa 3.6), and 3,5 bis(trifluoromethyl) (pKa 3.2).

Also, by analysing the data in Table 1 we can observe that, generally, the most active derivatives contain an alkoxy group with chain lengths ranging between 8 and 12 carbons.

Since it was previously suggested [9] that esters of weak acids may function by releasing an acid within mycobacterial cells, lowering the pH of the intracellular medium, the examination of the activity of esters derived from acids with similar pKa to the nitro-containing compounds was important. Esters made from acids with comparable pKa should have comparable antibacterial activity if that was the only mechanism of action. The differences observed by modifying the alkoxy group would be due to the relative rate of absorption and hydrolysis inside the target bacteria.

**Figure 1:**
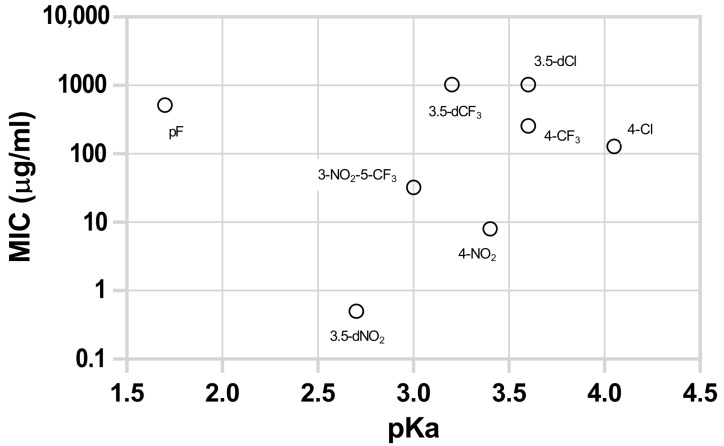
Antitubercular activity (MIC) against *M. tuberculosis* of eight decyl esters with different substituents in the benzoyl group in function of the pKa of the free acid liberated by hydrolysis. Aromatic substituents: 2,3,4,5,6-pentafluoro (pF), 3,5-dinitro (3,5-dNO_2_), 3-nitro-5-trifluoromethyl (3-NO_2_-5-CF_3_), 3,5-bis(trifluoromethyl) (3,5-dCF3), 4-nitro (4-NO_2_), 3,5-dichloro (3,5-dCl), 4-trifluoromethyl (4-CF_3_), and 4-chloro (4-Cl).

Figure 1 represents the MIC values of the several esters containing the same alkoxy group (C10) and different substitutions in the aromatic ring, yielding acids with different pKa upon hydrolysis. All nitro derivatives showed higher antitubercular activity than the rest of the tested compounds; however, 3,5-dinitro substituted derivatives showed a remarkably stronger effect. Compounds without nitro substitution presented lower activities, and no correlation between the pKa of the liberated acid and activity was observed. The most active non-nitro-substituted compound was the 4-chlorobenzoate, curiously the ester that yields a substituted acid with the highest pKa (4.0).

Noteworthily, the importance of correctly selecting the best alkoxy group is visible in all the derivatives, and more strikingly in the case of dodecyl-3-nitro-5-(trifluoromethyl)benzoate (52), which unexpectedly presented no bioactivity, while decyl and octyl-3-nitro-5-(trifluoromethyl)benzoate presented activity against *M. tuberculosis*, in line with what the corresponding 4-nitro substitution showed. A less striking example is compound **44**, which presented lower activity than would be expected given the remainder of the 3,5-dinitro series.

**Figure 2:**
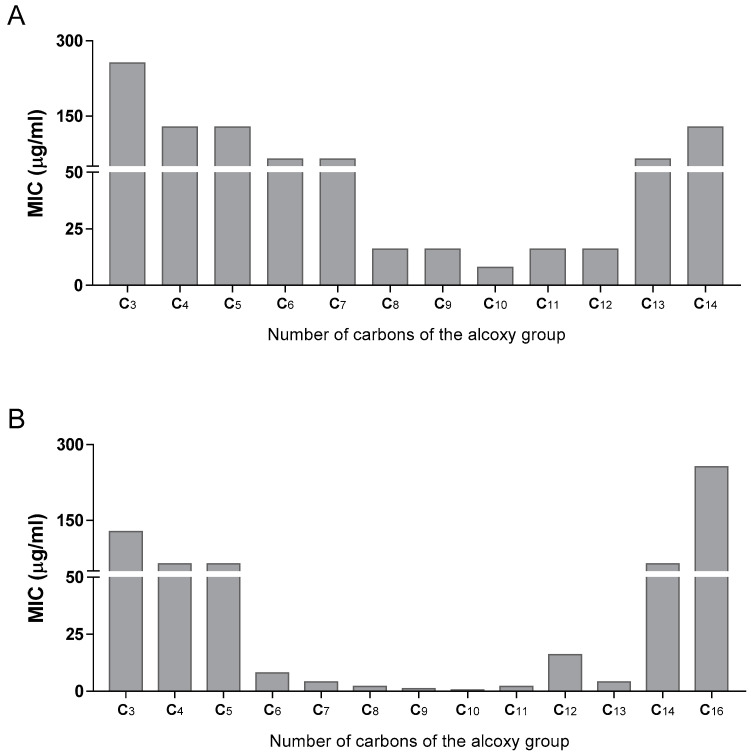
(MIC of 2 series of esters against *M. tuberculosis* in function of the chain length of the alkoxy group (**A**) 4-NO_2_ series, (**B**): 3,5-NO_2_ series). The numbers in the *X*-axis represent the carbon length of the alkyl chain. All alkyl chains are linear and saturated.

Next, for the most active compounds (4-nitro and 3,5-dinitro series), we studied the influence of the ester alkoxy group in the activity. Figure 2A,B shows how the activity of these derivatives can be modulated by selecting the alkoxy chain. The most active compounds were obtained with chain lengths between 8 and 12 carbons. Compounds **39**–**43** of the 3,5-dinitro series were especially active. The same pattern was seen in the 3-nitro-5-trifluoromethyl family as well (Table 1).

#### 3.1.2. Thioesters

Since the presence of the nitro group greatly influenced the activity of the compounds, we created a smaller series of analogous nitro thioesters to examine whether their bioactivity would follow the same trend, involving both the length of the alkyl chain and the aromatic substituents (Table 2).

**Figure 3:**
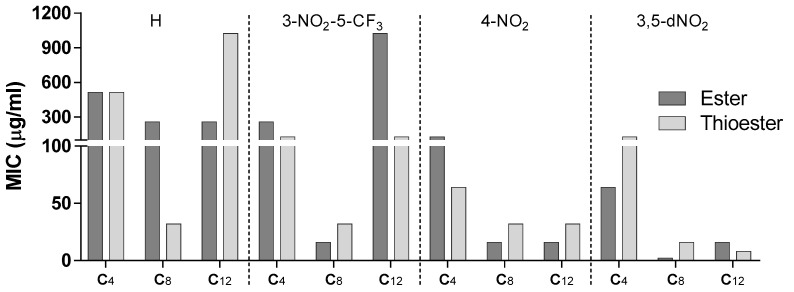
Comparison of the MIC of the esters and corresponding thioesters against *M. tuberculosis.* Each series has a different electron-withdrawing substituent in the aromatic ring and consists of the butyl (C4), octyl (C8), and dodecyl (C12) esters and corresponding thioesters. Aromatic substituents: hydrogen (H), 3-nitro-5-trifluoromethyl (3-NO_2_-5-CF_3_), 4-nitro (4-NO_2_), and 3,5-dinitro (3,5-NO_2_).

Figure 3 represents the comparison between the activity of esters and thioesters expressed as MIC (µg/mL).

From Figure 3, we can observe that isosteric substitution did not provide a meaningful advantage to the activity. If we look at the more active compounds (nitrated derivatives with C8-C12 chain lengths), we could not improve further the activity of the already more active compounds having C8 chains (compounds **29**, **39**, and **50**) and merely improve the activity of the C12 3,5-dinitro compound (**44**). However, as can be seen from Figure 2B, compound **44** (C12) was not a particularly active ester. The other situation where the isosteric substitution brought an immediate advantage was in the simple benzoate thioester with a C8 chain, that again, was not a particularly active ester.

Since one of the potential applications considered for esters of weak acids is their use as prodrugs, their stability in several media was determined, namely, in phosphate buffer adjusted to pH 7.4 (PBS), human plasma, and *Mycobacterium smegmatis* homogenate, as described in the methodologies section.

The PBS stability study is presented in Table 3. This study aimed at testing if the hydrolysis observed in biological media was due to chemical instability of the compounds or to enzymatic hydrolysis. For most compounds, the PBS stability assays showed very low chemical degradation, and the results are presented in percentage of degradation at 3 time points (1, 7, and 14 days).

In all compounds that presented degradation during stability incubations, the peak of free acid resulting from hydrolysis of esters and thioesters was the only product observable by HPLC.

Most of the compounds showed chemical stability in PBS (Table 3) that is compatible with the incubation time for MIC determination. Esters and thioesters that have shown the highest activity are stable during the incubation periods used in the assay. Some compounds with 4-carbon alkoxy or alkylthio groups have lower stabilities and can give rise to significant amounts of the free acid (>50%) by hydrolysis during MIC determination.

The remaining stability assays were performed in biological media. The compounds were incubated with either human plasma (HP) or mycobacterial homogenate (MH), and the obtained k_obs_ values are represented in Table 4.

Regarding plasma stability, it was observed that esters, and thioesters with no substitution in the aromatic ring, presented lower stability. The stability is, in general, dependent on the alkoxy or alkylthio chain length. Compounds with shorter alkyl chain lengths are hydrolysed faster than compounds with longer chains. The differences observed in the k_obs_ values for most esters with 8–12 carbon atoms chains are relatively small. For several derivatives with long alkoxy chains, no plasma degradation was observed. An interesting observation is that the compounds with higher mycobacterial activity have good stability in human plasma, which is a valuable property for future potential use.

We tested mycobacterial homogenate stability for a selected group of compounds of each nitro-substituted family, because it is important to determine if they are degraded by mycobacterial enzymes. Simple benzoates and thiobenzoates were also included to serve as a reference. The main observation is that the benzoates and thiobenzoates with no substitution on the aromatic ring are easily hydrolysed but presented lower activities. The compounds with higher activity (3,5-dinitrobenzoates with long-chain esters) were not degraded in the mycobacterial incubations. Other highly active compounds (4-nitrobenzoates and 3-nitro-5-(trifluoromethyl)benzoates) were hydrolysed at a low rate when compared to the simple benzoates and thiobenzoates.

### 3.2. Cytotoxicity

The metabolization of the nitro group is integrated into the mechanism of action of several new and old pharmaceutical compounds; however, it can also lead to the formation of toxic, genotoxic, mutagenic, and/or carcinogenic intermediates. Many nitro compounds can generate labile oxygen and nitrogen species that lead to reactions with biomolecules [24]. Since our most active compounds are nitro-substituted, and this functional group is highly associated with adverse effects, it was imperative to assess the toxicity of these compounds. Therefore, human monocytic cell line THP-1 (ATCC56 TIB202) was used, and the cytotoxicity results are expressed as the lethal concentration for 50% of the cell population, LC50 [25].

Table 5 provides a summary of the results obtained as well as a toxicity/activity index that was defined as the ratio between the LC50 and MIC values. This allows an easier assessment of what compounds can reach toxic levels at antitubercular active concentrations and, consequentially, what would be the most likely compounds to be acceptable as future drugs. The higher the T/A, the less likely the compound presents toxicity at relevant concentrations. For some compounds, the T/A index could not be calculated as the compounds showed no toxicity at the concentrations tested.

The results show that there is no relation between the toxicity and antimicrobial activity of the compounds. Given the traditional association between nitro-containing compounds and toxicity, one could expect the high antimicrobial activity of the nitro compounds to be associated with high toxicity. However, the more active compounds were not the most toxic. In fact, 3,5-dinitrobenzoate esters toxicity/activity index (T/A) is elevated, indicating that these would represent the preferred candidates for further study and application. Regarding the thioesters, with the limited comparisons between isosteres available (compounds **28** vs. **58**, **40** vs. **61** and **52** vs. **64**), no significant differences in toxicity were observed.

## 4. Discussion

The approach taken in the current study aimed at exploring the impact of several structural features in nitrobenzoates to achieve the best antimycobacterial lead compounds. Our findings revealed the more active compounds as those with an aromatic nitro substitution, the 3,5-dinitro esters being the most active series.

In the past, we proved that mycobacteria can hydrolyse esters to the corresponding acids, resulting in their activation [11,12]. An explanation for the activity of weak acids against Mtb is the acidification of the mycobacteria cytosol and the disruption of transmembrane transport systems [9,10].

Esters could act as transporters of the acids, but intracellular acidification is probably not the main driver for the activity of nitro compounds since we observed that esters containing these groups were much more active than analogues that yield acids with similar or lower pKa. When comparing the physical properties of the tested compounds, it is clear that the greater antitubercular effect observed for the nitro derivatives is unrelated to their LogP, pKa values, or hydrolysis rates. While chlorinated and nitrated derivatives can show similar pKa values for the corresponding acids, also with very similar hydrolysis rates, the bioactivity observed is widely different (ex: comparing compounds **16** vs. **31**, k_obs_ HP = 0.593 h^−1^ vs. 0,660 h^−1^; pKa = 3.6 vs. 3.4; MIC = 1024 µg/mL vs. 16 µg/mL, respectively).

There is precedent in the literature to the relevance of nitro moieties in the development of new compounds against Mtb. Nitrobenzoic acid is known to inhibit Mtb growth in vitro and has been used to identify *M. tuberculosis* complex species [26]. Additionally, molecules containing a dinitrobenzamide group like DNB1 are a new group of important candidates for antitubercular drugs (Dinitrobenzamides, DNBs), and are described as inhibitors of the enzyme decaprenylphosphoryl-beta-D-ribose oxidase (DprE1) [27,28,29]. DprE1 is a key flavoenzyme in Mtb, as it catalyses a critical step in the synthesis of arabinogalactan and lipoarabinomannan, which are vital compounds for the biosynthesis of the cell wall. [30,31]. This enzyme has periplasmic localization, which makes it an attractive target [32,33]. Many compounds with various scaffolds have been found to covalently or noncovalently inhibit DprE1 [34]. Covalent inhibition occurs by the formation of a nitroso group from the nitro group, which then specifically binds with the thiol group of the cys-387 residue present in the active centre of DprE1 [35,36]. The last ten years have seen a number of DprE1 inhibitor reviews that span both scaffold and docking studies [37,38,39,40]. Considering the activity results, we believe that the nitroaromatic moiety is the main driver of antimycobacterial activity. Our compounds are structurally similar to DNB1 and have the same dinitro acyl moiety [27], so DprE1 is an attractive target for the nitrobenzoate compounds here explored.

Other mechanisms of action for compounds containing nitro groups are possible and cannot be discarded at the moment. Another nitro-containing class of compounds especially relevant in the combat against TB is nitroimidazoles [41]. Delamanid and pretomanid belong to this class [42]. Although the exact mechanism of action of these two substances is not completely elucidated, it is believed to involve two distinct modes of action: non-specific respiratory poisoning and specific interference with mycolic acid synthesis [42,43]. These substances are considered to be prodrugs that need to be activated by the enzyme mycobacterial deazaflavin-dependent nitroreductase (Ddn) to produce reactive intermediates (yet to be identified in the case of delamanid) [42].

The alkoxy moiety of nitro-containing benzoates was extensively explored in the present study since the lipophilicity of these compounds could be an important factor for their absorption by mycobacteria, and, consequently, their antitubercular activity. Indeed, by comparing MIC values with LogP values (Table 1), it is observed that the bioactivity tends to increase with the lipophilicity of the compounds until around 10 carbon atoms in size, at which point the bioactivity starts declining with the increase in the lipophilicity. One must consider that, for these compounds, the increase in lipophilicity also leads to a decrease in solubility, and for compounds with alkyl chains greater than 10 carbon atoms in length, solubility issues in biological media were observed. Hence, it is plausible that greater lengths of alkyl chain possess the potential for similar if not greater antitubercular activity but are hindered by their solubility.

Since in the past it was proposed that the esters were merely prodrugs of the corresponding weak acids (simple benzoates), we should not discard that the same is applicable to this context. However, the most active compounds are not hydrolysed in mycobacterial homogenate and are stable in phosphate-buffered saline solution (PBS), indicating that in the assay conditions they are acting as drugs and not as prodrugs, as previously hypothesized. Moreover, in the past we compared the activity of the free acids and esters of 4-nitrobenzoates and 3,5-dinitrobenzoates, and we found that the activity of the esters was greater than the corresponding free acids [15], which supports the intrinsic activity of the esters.

Isosteric esters and thioesters give rise to the same free acid but have different hydrolysis rates and most probably different affinities for a putative target. If the compounds were acting as prodrugs, then it would be expected that the isosteric 3,5-dinitrothioesters showed enhanced activity compared to the esters. In the present study, it was observed that the thioesters are hydrolysed in mycobacterial homogenate, while we did not observe hydrolysis of the related esters. However, we did not observe an improved activity in the thioesters. As an example, thioester 60 presented a MIC of 16 µg/mL, while the corresponding ester (39) presented a MIC of 2 µg/mL. The thioester was hydrolysed with a k_obs_ of 79 h^−1^, while the ester did not show hydrolysis in the mycobacterial homogenate. On the other hand, if the compounds are acting as drugs, then the poorer interaction of the thioester with the target might be the relevant factor for antitubercular activity.

**Figure 4:**
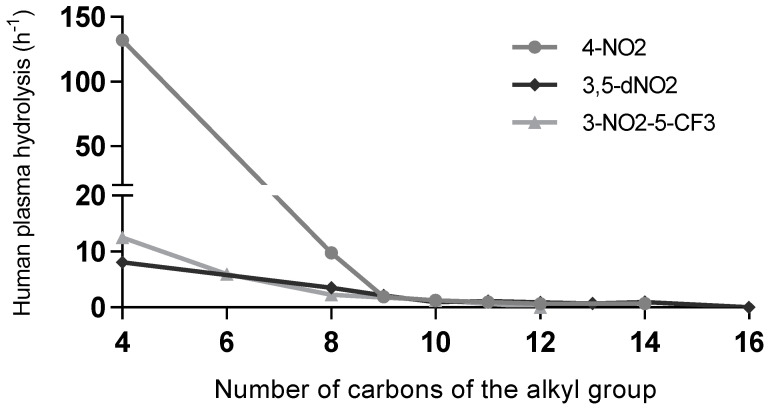
Stability in plasma of derivatives with nitro groups in the aromatic ring as a function of the chain lengths. Aromatic substituents: 4-nitro (4-NO_2_), 3,5-dinitro (3,5-dNO_2_), and 3-nitro-5-(trifluoromethyl) (3-NO_2_-5-CF_3_).

Plasma stability is less relevant for the activity discussion than mycobacterial homogenate stability, since activity tests were not conducted in plasma. However, hydrolysis by plasma esterases is also not related to activity, demonstrating once more that in addition to having a low affinity for mycobacterial esterases, the more active compounds also have a low affinity for plasma esterases regarding hydrolysis. Comparing the bioactivity (MIC values) with the stability in human plasma (k_obs_ values) becomes relevant if one considers future possible applications of such compounds. Esters are considered very labile functional groups, and human plasma has relevant esterase activity; however, the most active compounds show a reduced hydrolysis rate (Figure 4), with only the short-chain derivatives being easily hydrolysed in human plasma. The longer alkyl chains show very low hydrolysis rates.

The strong antimicrobial activity of nitro compounds might be related to the high toxicity given the traditional association between nitro-containing chemicals and toxicity, yet the more active compounds were not the most toxic. In fact, 3,5-dinitrobenzoate esters have a high toxicity/activity index (T/A), making them candidates for additional research and application. We plan to further develop this class of compounds by exploring other isosteric structures that improve the stability of the molecules and may show greater activity. Since amides are known to be stable regarding esterase hydrolysis and are also structurally related to known inhibitors of DprE1, we plan to further develop this line of research by adding several nitro-substituted *N*-alkylbenzamides.

## Figures and Tables

**Table 1:** Structure, physical properties and antitubercular activity of benzoate esters against Mtb H37Rv.

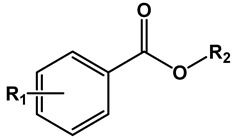							
Compound	R_1_	R_2_	MW (g/mol)	pKa *	LogP **	MIC (μg/mL)	MBC (μg/mL)
Esters							
**1**	H	-CH_2_(CH_2_)_2_CH_3_	178.23	4.1	3.40	>512	>512
**2**		-CH_2_(CH_2_)_4_CH_3_	206.29		4.51	512	512
**3**		-CH_2_(CH_2_)_6_CH_3_	234.35		5.47	256	512
**4**		-CH_2_(CH_2_)_10_CH_3_	290.45		7.16	256	512
**5**	4-Cl	-CH_2_(CH_2_)_7_CH_3_	282.81	4.0	6.42	64	256
**6**		-CH(CH_3_)(CH_2_)_6_CH_3_	282.81		6.62	128	512
**7**		-CH_2_(CH_2_)_8_CH_3_	296.84		6.86	128	256
**8**		-CH_2_(CH_2_)_9_CH_3_	310.86		7.30	1024	>1024
**9**		-CH_2_(CH_2_)_10_CH_3_	324.89		7.70	>1024	>1024
**10**		-CH_2_(CH_2_)_12_CH_3_	352.94		8.52	>1024	>1024
**11**		-CH_2_(CH_2_)_14_CH_3_	381.00		9.25	1024	1024
**12**	3,5-dCl	-CH_2_(CH_2_)_7_CH_3_	317.25	3.6	6.87	>1024	>1024
**13**		-CH(CH_3_)(CH_2_)_6_CH_3_	317.25		7.17	1024	>1024
**14**		-CH_2_(CH_2_)_8_CH_3_	331.28		7.33	>1024	>1024
**15**		-CH_2_(CH_2_)_9_CH_3_	345.30		7.69	>1024	>1024
**16**		-CH_2_(CH_2_)_10_CH_3_	359.33		8.10	1024	1024
**17**		-CH_2_(CH_2_)_12_CH_3_	387.38		8.93	1024	1024
**18**	4-CF_3_	-CH_2_(CH_2_)_8_CH_3_	330.39	3.6	6.45	256	1024
**19**	3,5-dCF_3_	-CH_2_(CH_2_)_8_CH_3_	398.39	3.2	6.46	>1024	>1024
**20**	2,3,4,5,6-pF	-CH_2_(CH_2_)_8_CH_3_	352.35	1.7	5.42	512	>1024
**21**	4-NO_2_	-CH_2_CH_2_CH_3_	209.20	3.4	2.58	256	256
**22**		-CH_2_(CH_2_)_2_CH_3_	223.23		2.83	128	256
**23**		-CH_2_(CH_2_)_3_CH_3_	237.26		3.25	128	128
**24**		-CH_2_(CH_2_)_4_CH_3_	251.28		3.73	64	128
**25**		-CH_2_(CH_2_)_5_CH_3_	265.31		4.20	64	128
**26**		-CH_2_(CH_2_)_6_CH_3_	279.34		4.69	16	64
**27**		-CH_2_(CH_2_)_7_CH_3_	293.36		5.22	16	64
**28**		-CH(CH_3_)(CH_2_)_6_CH_3_	293.36		5.58	16	64
**29**		-CH_2_(CH_2_)_8_CH_3_	307.39		5.63	8	16
**30**		-CH_2_(CH_2_)_9_CH_3_	321.41		6.09	16	64
**31**		-CH_2_(CH_2_)_10_CH_3_	335.44		6.61	16	128
**32**		-CH_2_(CH_2_)_11_CH_3_	349.47		7.07	64	128
**33**		-CH_2_(CH_2_)_12_CH_3_	363.50		7.59	128	512
**34**	3,5-dNO_2_	-CH_2_CH_2_CH_3_	254.20	2.7	2.40	128	256
**35**		-CH_2_(CH_2_)_2_CH_3_	268.23		2.77	64	256
**36**		-CH_2_(CH_2_)_3_CH_3_	282.26		3.06	64	128
**37**		-CH_2_(CH_2_)_4_CH_3_	296.28		3.57	8	64
**38**		-CH_2_(CH_2_)_5_CH_3_	310.31		4.05	4	16
**39**		-CH_2_(CH_2_)_6_CH_3_	324.34		4.41	2	8
**40**		-CH_2_(CH_2_)_7_CH_3_	338.36		4.74	1	4
**41**		-CH(CH_3_)(CH_2_)_6_CH_3_	338.36		5.23	0.5	1
**42**		-CH_2_(CH_2_)_8_CH_3_	352.39		5.18	0.5	2
**43**		-CH_2_(CH_2_)_9_CH_3_	366.41		5.73	2	4
**44**		-CH_2_(CH_2_)_10_CH_3_	380.44		6.08	16	32
**45**		-CH_2_(CH_2_)_11_CH_3_	394.47		6.33	4	512
**46**		-CH_2_(CH_2_)_12_CH_3_	408.50		6.62	64	256
**47**		-CH_2_(CH_2_)_14_CH_3_	436.55		7.27	256	256
**48**	3-NO_2_-5-CF_3_	-CH_2_(CH_2_)_2_CH_3_	291.23	3.0	3.53	256	1024
**49**		-CH_2_(CH_2_)_4_CH_3_	319.28		4.34	64	1024
**50**		-CH_2_(CH_2_)_6_CH_3_	347.34		4.97	16	64
**51**		-CH_2_(CH_2_)_8_CH_3_	375.39		5.86	32	256
**52**		-CH_2_(CH_2_)_10_CH_3_	403.44		6.79	>1024	>1024
INH ***						0.0625	0.125

* pKa values refer to the respective aromatic acid moiety that would be the product of its hydrolysis. Values were estimated using the MolGpKa webserver [19]. Experimental pKa values obtained from the literature are: benzoic acid: 4.204 [20], 4-chlorobenzoic acid: 3.98 [21], 3,5-dichlorobenzoic acid: 3.10 [22], 4-nitrobenzoic acid: 3.42, pentafluorobenzoic acid: 1.75 [20], 3,5-dinitrobenzoic acid: 2.67 [22]. ** LogP values were predicted by the software ALOGPS 2.1 [23]. *** Isoniazid (INH) was used as the positive control in antimycobacterial assays.

**Table 2:** Structure, physical properties, and antitubercular activity of benzoate thioesters against Mtb H37Rv.

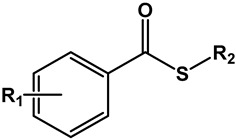							
Compound	R_1_	R_2_	MW (g/mol)	pKa *	LogP **	MIC (μg/mL)	MBC (μg/mL)
**Thioesters**							
**53**	H	-CH_2_(CH_2_)_2_CH_3_	194.29	4.1	3.89	512	>1024
**54**		-CH_2_(CH_2_)_6_CH_3_	250.40		5.43	32	256
**55**		-CH_2_(CH_2_)_10_CH_3_	306.50		7.07	1024	>1024
**56**	4-NO_2_	-CH_2_(CH_2_)_2_CH_3_	239.29	3.4	3.68	64	128
**57**		-CH_2_(CH_2_)_6_CH_3_	295.40		5.16	32	128
**58**		-CH_2_(CH_2_)_10_CH_3_	351.50		6.80	32	512
**59**	3,5-dNO_2_	-CH_2_(CH_2_)_2_CH_3_	284.29	2.7	2.88	128	256
**60**		-CH_2_(CH_2_)_6_CH_3_	340.40		4.55	16	64
**61**		-CH_2_(CH_2_)_10_CH_3_	396.50		6.13	8	32
**62**	3-NO_2_-5-CF_3_	-CH_2_(CH_2_)_2_CH_3_	307.29	3.0	3.59	128	512
**63**		-CH_2_(CH_2_)_6_CH_3_	363.40		5.13	32	128
**64**		-CH_2_(CH_2_)_10_CH_3_	419.50		6.79	128	512
INH ***						0.0625	0.125

* pKa values refer to the respective aromatic acid moiety that would be the product of its hydrolysis. Values were estimated using the software MolGpKa [19]. Experimental pKa values obtained from the literature are: benzoic acid: 4.204 [20], 4-nitrobenzoic acid: 3.42, and 3,5-dinitrobenzoic acid: 2.67 [22]. ** LogP values were predicted by the software ALOGPS 2.1 [23]. *** Isoniazid (INH) was used as the positive control in antimycobacterial assays.

**Table 3:** Results obtained from stability studies in phosphate buffer (PBS). Results presented as percentage (%) of hydrolysis observed at each time point.

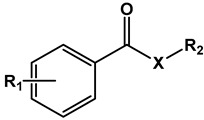					
Compound	R_1_	R_2_	PBS (1 Day)	PBS (7 Days)	PBS (14 Days)
**Esters (X = O)**					
**1**	H	-CH_2_(CH_2_)_2_CH_3_	0	1	2
**3**		-CH_2_(CH_2_)_6_CH_3_	0	0	0
**4**		-CH_2_(CH_2_)_10_CH_3_	0	0	0
**22**	4-NO_2_	-CH_2_(CH_2_)_2_CH_3_	0	2	4
**26**		-CH_2_(CH_2_)_6_CH_3_	0	3	6
**31**		-CH_2_(CH_2_)_10_CH_3_	0	1	1
**35**	3,5-dNO_2_	-CH_2_(CH_2_)_2_CH_3_	10	40	65
**39**		-CH_2_(CH_2_)_6_CH_3_	0	2	3
**44**		-CH_2_(CH_2_)_10_CH_3_	0	1	2
**48**	3-NO_2_-5-CF_3_	-CH_2_(CH_2_)_2_CH_3_	5	35	60
**50**		-CH_2_(CH_2_)_6_CH_3_	0	3	6
**52**		-CH_2_(CH_2_)_10_CH_3_	1	6	11
**Thioesters (X = S)**					
**53**	H	-CH_2_(CH_2_)_2_CH_3_	0	0	0
**54**		-CH_2_(CH_2_)_6_CH_3_	0	0	1
**55**		-CH_2_(CH_2_)_10_CH_3_	0	0	0
**56**	4-NO_2_	-CH_2_(CH_2_)_2_CH_3_	0	2	4
**57**		-CH_2_(CH_2_)_6_CH_3_	0	0	1
**58**		-CH_2_(CH_2_)_10_CH_3_	0	0	0
**59**	3,5-dNO_2_	-CH_2_(CH_2_)_2_CH_3_	3	19	35
**60**		-CH_2_(CH_2_)_6_CH_3_	0	1	3
**61**		-CH_2_(CH_2_)_10_CH_3_	0	0	1
**62**	3-NO_2_-5-CF_3_	-CH_2_(CH_2_)_2_CH_3_	1	10	18
**63**		-CH_2_(CH_2_)_6_CH_3_	0	2	3
**64**		-CH_2_(CH_2_)_10_CH_3_	0	0	0

**Table 4:** Results obtained from stability studies in human plasma (HP) and mycobacterial homogenate (MH). Results presented as k_obs_ (×100 h^−1^).

Compound	k_obs_ HP (×100 h^−1^)	k_obs_ MH (×100 h^−1^)	Compound	k_obs_ HP (×100 h^−1^)	k_obs_ MH (×100 h^−1^)
**1**	44.1 ± 6.1	356 ± 17	**40**	2.140 ± 0.191	
**3**	2.66 ± 0.23	35.5 ± 0.3	**41**	0.869 ± 0.019	n/o
**4**	n/o	0.76 ± 0.02	**42**	0.98 ± 0.07	n/o
**5**	1.66 ± 0.025		**43**	1.130 ± 0.061	
**6**	0.387 ± 0.025		**44**	0.89 ± 0.13	n/o
**7**	1.05 ± 0.061		**45**	0.724 ± 0.020	
**8**	0.899 ± 0.032		**46**	0.97 ± 0.23	n/o
**9**	0.806 ± 0.010		**47**	n/o	n/o
**10**	0.682 ± 0.013		**48**	12.5 ± 1.8	13.0 ± 1.1
**11**	n/o		**49**	5.96 ± 1.28	0.67 ± 0.06
**12**	1.04 ± 0.030		**50**	2.30 ± 0.06	n/o
**13**	0.391 ± 0.021		**51**	1.26 ± 0.04	n/o
**14**	0.796 ± 0.023		**52**	n/o	n/o
**15**	0.676 ± 0.034		**Thioesters**		
**16**	0.593 ± 0.026		**53**	92.3 ± 1.8	417 ± 10
**17**	n/o		**54**	14.9 ± 0.9	26.9 ± 1.9
**22**	132 ± 6.0	20.4 ± 3.3	**55**	0.60 ± 0.07	0.79 ± 0.08
**26**	9.73 ± 1.2	1.80 ± 0.38	**56**	34.5 ± 1.3	11.3 ± 0.8
**27**	1.87 ± 0.027		**57**	8.08 ± 0.21	3.20 ± 0.11
**28**	0.800 ± 0.058		**58**		
**29**	1.30 ± 0.061		**59**	10.3 ± 1.9	0.74 ± 0.03
**30**	0.939 ± 0.053		**60**	11.8 ± 1.9	0.79 ± 0.02
**31**	0.660 ± 0.10	0.88 ± 0.05	**61**	2.85 ± 0.21	0.38 ± 0.02
**33**	0.703 ± 0.042		**62**	7.86 ± 0.64	0.32 ± 0.03
**35**	8.08 ± 0.29	11.0 ± 0.8	**63**	3.47 ± 0.54	0.40 ± 0.02
**39**	3.53 ± 0.44	n/o	**64**	0.78 ± 0.01	0.15 ± 0.01

n/o—hydrolysis not observed.

**Table 5:** Results obtained from cytotoxicity assays, represented by LC50 values and its comparison with MIC values via a toxicity/activity (T/A) index.

Compound	MIC (μg/mL)	LC50 (μg/mL)	T/A	Compound	MIC (μg/mL)	LC50 (μg/mL)	T/A
**5**	64	NT	NA	**34**	128	571	4.46
**6**	128	600	4.68	**35**	64	456	7.13
**7**	128	350	2.73	**36**	64	138	2.16
**8**	1024	653	0.638	**37**	8	528	66.0
**9**	>1024	624	>0.609	**38**	4	1137	284
**10**	>1024	461	>0.450	**39**	2	NT	NA
**11**	1024	NT	NA	**40**	1	875	875
**12**	>1024	151	>0.147	**41**	1	561	561
**13**	1024	345	0.337	**42**	0.5	NT	NA
**14**	>1024	144	>0.141	**43**	2	515	257
**15**	>1024	690	>0.673	**44**	16	NT	NA
**16**	1024	516	0.504	**45**	4	184	46.0
**17**	1024	421	0.411	**46**	64	NT	NA
**21**	256	NT	NA	**47**	256	NT	NA
**22**	128	NT	NA	**48**	256	552	2.16
**23**	128	NT	NA	**49**	64	639	9.98
**24**	64	438	6.85	**50**	16	NT	NA
**25**	64	681	10.6	**51**	32	NT	NA
**26**	16	869	54.3	**52**	>1024	NT	NA
**27**	16	1217	76.1	**58**	32	NT	NA
**28**	16	1174	73.4	**61**	8	NT	NA
**29**	8	103	12.8	**64**	128	513	4.00
**30**	16	51.1	3.19				
**31**	16	623	38.9				
**32**	64	417	6.51				
**33**	128	487	3.80				

NT—no toxicity observed. NA—not applicable as no toxicity was observed.

## Data Availability

Data available within the article or its Supplementary Materials.

## Supplementary Materials

The following supporting information can be downloaded at: https://www.mdpi.com/article/10.3390/microorganisms11040969/s1, Annex 1—Structural characterization.
