# Supplementary material for: Nitrobenzoates and Nitrothiobenzoates with Activity against M. tuberculosis
Source: Microorganisms. 2023 Apr 8;11(4):969. doi: 10.3390/microorganisms11040969 (PMC10142844; doi:10.3390/microorganisms11040969)
Supplement: Supplementary file 1 [file microorganisms-11-00969-s001.zip › microorganisms-2280350-supplementary.pdf]

Annex 1 – Structural characterization:

Dodecyl benzoate (4). Yield: 72%; **^{1}H NMR (300 MHz, CDCl$_3$): δ** 8.11 – 8.01 (m, 2H), 7.62 – 7.53 (m, 1H), 7.53 – 7.39 (m, 2H), 4.33 (t, J = 6.7 Hz, 3H), 1.84 – 1.71 (m, 2H), 1.36 – 1.25 (m, 24H), 0.88 (t, J = 7.4 3H); **^{13}C NMR (100 MHz, CDCl$_3$): δ** 166.88, 132.76, 130.71, 129.53, 128.30, 65.34, 31.17, 26.00, 14.10; **MS (ESI$^+$):** *m/z* calculated for $C_{19}H_{30}O_2$: 290.440, found: 291.4 (M+H$^+$)

Nonyl 4-chlorobenzoate (**5**). Yield: 74%; **^{1}H NMR (300 MHz, CDCl$_3$): δ** 7.98 (*d, J*= 3Hz, 2H), 7.41 (*d, J*= 3Hz, 2H), 4.31 (*t, J*= 6Hz, 2H), 1.76 *m,* 2H), 1.33 – 1.11 (*m,* 12H), 0.88 (*t,* J= 6Hz, 3H); **^{13}C NMR (100 MHz, CDCl$_3$): δ** 165.77, 139.21, 130.93, 128.95, 128.64, 66.38, 31.86, 29.49, 29.29, 29.25, 29.68, 26.02, 22.68, 14.15; **MS (ESI$^+$):** *m/z* calculated for $C_{16}H_{23}ClO_2$: 282.805, found: 283.7 (M+H$^+$)

1-Methyloctyl 4-chlorobenzoate (**6**) Yield: 93%; **^{1}H NMR (300 MHz, CDCl$_3$): δ** 7.99 (*d, J*= 3Hz, 2H), (*d, J*= 3Hz, 2H), 5.16 (*m,* 1H), 1.72 (*m,* 1H), 1.63 (*m,* 1H), 1.37 (*d, J*=12Hz, 3H), 1.49 – 1.28 (*m,* 10H), 0.88 (*t, J*= 6Hz, 3H); **^{13}C NMR (100 MHz, CDCl$_3$): δ** 165.99, 138.45, 135.09, 130.75, 129.99, 64.70, 33.98, 30.61, 29.89, 29.71, 29.58, 29.34, 21.68, 15.9, 14.15; **MS (ESI$^+$):** *m/z* calculated for $C_{16}H_{23}ClO_2$: 282.805, found: 283.9 (M+H$^+$)

Decyl 4-chlorobenzoate (**7**) Yield: 62%; **^{1}H NMR (300 MHz, CDCl$_3$): δ** 7.99 (*d, J*= 3Hz, 2H), 7.42 (*d, J*= 3Hz, 2H), 4.32 (*t, J*= 6Hz, 2H), 1.77 (*m,* 2H), 1.44–1.28 (*m,* 14H), 0.89 (*t, J*= 6Hz, 6H); **^{13}C NMR (100 MHz, CDCl$_3$): δ** 165.79, 139.22, 130.93, 128.97, 128.65, 65.43, 31.94, 29.67, 29.59, 29.54, 29.38, 28.67, 26.03, 22.71, 14.15; **MS (ESI$^+$):** *m/z* calculated for $C_{17}H_{25}ClO_2$: 296.832, found: 297.9 (M+H$^+$)

Undecyl 4-chlorobenzoate (**8**) Yield: 95%; **^{1}H NMR (300 MHz, CDCl$_3$): δ** 7.99 (*d, J*= 3Hz, 2H), 7.42 (*d, J*= 3Hz, 2H), 4.32 (*t, J*= 6Hz, 2H), 1.77 (*m,* 2H), 1.43 – 1.27 (*m,* 16H), 0.88 (*t, J*= 6Hz, 3H); **^{13}C NMR (100 MHz, CDCl$_3$): δ** 165.79, 139.22, 130.93, 128.87, 128.55, 66.40, 31.91, 29.61, 29.59, 29.53, 29.34, 29.28, 28.68, 26.02, 22.69, 14.12; **MS (ESI$^+$):** *m/z* calculated for $C_{18}H_{27}ClO_2$: 310.858, found: 311.8 (M+H$^+$)

Dodecyl 4-chlorobenzoate (**9**) Yield: 83%; **^{1}H NMR (300 MHz, CDCl$_3$): δ** 7.99 (*d,* J= 3Hz, 2H), 7.43 (*d, J*= 3Hz, 2H), 4.32 (*t, J*= 6Hz, 2H), 1.75 (*m,* 2H), 1.44 – 1.10 (*m,* 18H), 0.90 (*t, J*= 6Hz, 3H); **^{13}C NMR (100 MHz, CDCl$_3$): δ** 165.83, 139.23, 130.94, 128.99, 128.67, 65.41, 31.92, 29.64, 29.52, 29.35, 29.37, 28.08, 26.02, 22.69, 14.12; **MS (ESI$^+$):** *m/z* calculated for $C_{19}H_{29}ClO_2$: 324.885, found: 325.8 (M+H$^+$)

Tetradecyl 4-chlorobenzoate (**10**) Yield: 87%, **^{1}H NMR (300 MHz, CDCl$_3$): δ** 7.90 (*d, J*= 3Hz, 2H), 7.35 (*d, J*= 3Hz, 2H), 4.23 (*t, J*= 6Hz, 2H), 1.69 (*m,* 2H), 1.33 – 1.18 (*m,* 22H), 0.81 (*t, J*= 9Hz, 3H), **^{13}C NMR (100 MHz, CDCl$_3$): δ** 165.85, 139.24, 130.95, 128.96, 128.68, 66.43, 31.94, 29.71, 29.67, 29.59, 29.54, 29.38, 29.28, 28.67, 26.02, 22.71, 14.15; **MS (ESI$^+$):** *m/z* calculated for $C_{21}H_{33}ClO_2$: 352.938, found: 353.9 (M+H$^+$)

Hexadecyl 4-chlorobenzoate (**11**) Yield: 87%, **^{1}H NMR (300 MHz, CDCl$_3$): δ** 7.90 (*d, J*= 3Hz, 2H), 7.35 (*d, J*= 3Hz, 2H), 4.23 (*t, J*= 6Hz, 2H), 1.69 (*m,* 2H), 1.33 – 1.18 (*m,* 26H), 0.81 (*t, J*= 9Hz, 3H), **^{13}C NMR (100 MHz, CDCl$_3$): δ** 165.85, 139.24, 130.95, 128.96, 128.68, 65.43, 31.94, 31.11, 30.84, 30.59, 30.03, 29.83, 29.74, 29.59, 29.23, 29.28, 29.01, 28.67, 26.02, 22.71, 14.15; **MS (ESI$^+$):** *m/z* calculated for $C_{23}H_{37}ClO_2$: 380.991, found: 382.0 (M+H$^+$)

Nonyl 3,5-dichlorobenzoate (**12**) Yield: 68%; **^{1}H NMR (300 MHz, CDCl$_3$): δ** 7.90 (*d, J*= 2Hz, 2H), 7.54 (*t, J*= 2Hz, 1H), 4.32 (*t, J*= 7Hz, 2H), 1.85-1.69 (*m,* 2H), 1.47 – 1.19 (*m,* 12H), 0.88 (*t, J*= 9Hz, 3H); **^{13}C NMR (100 MHz, CDCl$_3$): δ** 164.98, 135.65, 133.46, 130.96, 128.66, 66.17, 31.85, 29.61, 29.38, 28.77, 26.12, 22.69, 14.32; **MS (ESI$^+$):** *m/z* calculated for $C_{16}H_{22}Cl_2O_2$: 317.250, found: 318.2 (M+H$^+$)

1-Methyloctyl 3,5-dichlorobenzoate (**13**) Yield: 39%; **^{1}H NMR (300 MHz, CDCl$_3$): δ** 7.91 (*d, J*= 3Hz, 2H), 7.54 (*t, J*= 3Hz, 1H), 5.16 (*m,* 1H), 1.73 (*m,* 1H), 1.69 (*m,* 1H), 1.35 (*d, J*= 3Hz, 3H), 1.36 – 1.29 (*m,* 10H), 0.89 (*t, J*= 9Hz, 3H); **^{13}C NMR (100 MHz, CDCl$_3$): δ** 165.99, 138.55, 135.09, 131.05, 130.02,

64.71, 33.95, 31.11, 29.99, 29.81, 29.58, 29.44, 21.98, 15.9, 14.131; **MS (ESI$^+$):** *m/z* calculated for $C_{16}H_{22}Cl_2O_2$: 317.250, found: 318.3 (M+H$^+$)

Decyl 3,5-dichlorobenzoate (**14**) Yield: 62%; **^{1}H NMR (300 MHz, $CDCl_3$): δ** 7.90 (*d*, *J*= 2Hz, 2H), 7.54 (*t*, *J* = 2Hz, 1H), 4.32 (*t*, *J*= 6Hz, 2H), 1.85 - 1.65 (*m*, 2H), 1.50 – 1.18 (*m*, 14H), 0.88 (*t*, *J*= 9Hz, 3H); **^{13}C NMR (100 MHz, $CDCl_3$): δ** 164.48, 135.39, 133.49, 132.80, 128.13, 66.17, 32.04, 29.67, 29.44, 29.39, 28.74, 26.10, 22.82, 14.20; **MS (ESI$^+$):** *m/z* calculated for $C_{17}H_{24}Cl_2O_2$: 331.277, found: 332.4 (M+H$^+$)

Undecyl 3,5-dichlorobenzoate (**15**) Yield: 75%, **^{1}H NMR (300 MHz, $CDCl_3$): δ** 7.83 (*d*, *J*= 6Hz, 2H), 7.54 (*t*, *J* = 3Hz, 1H), 4.33 (*t*, *J*= 6Hz, 2H), 1.78 (*m*, 2H), 1.42 – 1.12 (*m*, 16H), 0.89 (*t*, *J*= 9Hz, 3H); **^{13}C NMR (100 MHz, $CDCl_3$): δ** 165.23, 134.23, 130.32, 129.50, 127,99, 67.78, 38.89, 31.92, 29.61, 29.35, 29.35, 29.26, 28.59, 25.96, 22.70, 14.13; **MS (ESI$^+$):** *m/z* calculated for $C_{18}H_{26}Cl_2O_2$: 345.303, found: 346.4 (M+H$^+$)

Dodecyl 3,5-dichlorobenzoate (**16**) Yield: 82%, **^{1}H NMR (300 MHz, $CDCl_3$): δ** 7.90 (*d*, *J*= 6Hz, 2H), 7.55 (*t*, *J* = 3Hz, 1H, C4-*H*), 4.32 (*t*, *J*= 6Hz, 2H), 1.76 (*m*, 2H), 1.39 – 1.20 (*m*, 18H), 0.88 (*t*, *J*= 6Hz, 3H); **^{13}C NMR (100 MHz, $CDCl_3$): δ** 164.36, 135.23, 132.66, 131.91, 127.99, 66.05, 31.93, 29.65, 29.65, 29.59, 29.51, 29.39, 29.26, 28.59, 25.96, 22.71, 14.15; **MS (ESI$^+$):** *m/z* calculated for $C_{19}H_{28}Cl_2O_2$: 359.330, found: 360.3 (M+H$^+$)

Tetradecyl 3,5-dichlorobenzoate (**17**) Yield: 83%, **^{1}H NMR (300 MHz, $CDCl_3$): δ** 7.90 (*d*, *J*= 6Hz, 2H), 7.73 (*t*, *J*= 3Hz, 1H), 4.32 (*t*, *J*= 6Hz, 2H), 1.76 (*m*, 2H), 1.40 – 1.19 (*m*, 22H), 0.87 (*t*, *J*= 9Hz, 3H); **^{13}C NMR (100 MHz, $CDCl_3$): δ** 164.34, 135.25, 133.30, 130.14, 127.99, 66.04, 31.94, 29.71, 29.66, 29.59, 29.51, 29.39, 29.26, 28.59, 25.96, 22.71, 14.15; **MS (ESI$^+$):** *m/z* calculated for $C_{21}H_{32}Cl_2O_2$: 387.383, found: 388.3 (M+H$^+$)

Decyl 4-(trifluoromethyl)benzoate **(18)** Yield: 89%, **^{1}H NMR (300 MHz, $CDCl_3$): δ** 8.15 (*d*, *J*= 9 Hz, 2H), 7.70 (*d*, *J*= 9 Hz, 2H), 4.34 (*t*, *J*= 6; 9 Hz, 2H), 1.71-1.84 (*m*, 2H), 1.20-1.49 (*m*, 14H), 0.87 (*t*, *J*= 6 Hz, 3H), **^{13}C NMR (100 MHz, $CDCl_3$): δ** 165.62, 135.09, 133.83, 130.08, 125.54, 124.61, 65.88, 32.03, 29.67, 29.45, 29.40, 28.77, 26.14, 22.83, 14.26; **MS (ESI$^+$):** *m/z* calculated for $C_{18}H_{25}F_3O_2$: 330.385, found: 331.4 (M+H$^+$)

Decyl 3,5-bis(trifluoromethyl)benzoate **(19)** Yield: 51%, **^{1}H NMR (300 MHz, $CDCl_3$): δ** 8.48 (*sa*, 2H), 8.06 (*sa*, **1H**), 4.39 (*t*, J= 6 Hz, 2H), 1.74-1.80 (*m*, 2H), 1.20-1.50 (m, 14H), 0.87 (*t*, J= 6 Hz, 3H), **^{13}C NMR (100 MHz, $CDCl_3$): δ** 164.01, 132.34, 131.89, 129.69, 126.25, 124.25, 65.44, 31.88, 29.24, 29.31, 28.57, 25.92, 22.68, 14.12; **MS (ESI$^+$):** *m/z* calculated for $C_{19}H_{24}F_6O_2$: 398.383, found: 399.5 (M+H$^+$)

Decyl 2,3,4,5,6-pentafluorobenzoate **(20)** Yield: 9%, **^{1}H NMR (300 MHz, $CDCl_3$): δ** 4.38 (*t*, *J*= 6; 9 Hz, 2H), 1.69-1.82 (*m*, 2H), 1.20-1.48 (*m*, 14H), 0.88 (*t*, *J*= 6 Hz, 3H), **^{13}C NMR (100 MHz, $CDCl_3$): δ** 165.83, 154.72, 145.44, 139.87, 109.88, 65.33, 28.78, 26.04, 29.38, 29.59, 29.67, 29.67, 31.95, 22.72, 14.12; **MS (ESI$^+$):** *m/z* calculated for $C_{17}H_{21}F_5O_2$: 352.339, found: 353.4 (M+H$^+$)

Butyl 4-nitrobenzoate **(22)** Yield: 89%, **^{1}H NMR (300 MHz, $CDCl_3$): δ** 8.28 (*dt*, J_o= 9; J_m= 1.8; 2.1 Hz, 2H), 8.20 (*dt*, J_o= 9; J_m= 1.8; 2.1 Hz, 2H), 4.36 (*t*, *J*= 6; 9 Hz, 2H), 1.71-1.85 (*m*, 2H), 1.41-1.55 (*m*, 2H), 0.99 (*t*, *J*= 6; 9 Hz, 3H); **^{13}C NMR (100 MHz, $CDCl_3$): δ** 164.89, 150.64, 136.04, 130.79, 123.65, 65.95, 30.78, 19.35, 13.84; **MS (ESI$^+$):** *m/z* calculated for $C_{11}H_{13}NO_4$: 223.225, found: 224.2 (M+H$^+$)

Pentyl 4-nitrobenzoate **(23)** Yield: 80%, **^{1}H NMR (300 MHz, $CDCl_3$): δ** 8.29 (*dt*, J_o= 9; J_m= 1.8; 2.1 Hz, 2H), 8.21 (*dt*, J_o= 9; J_m= 1.8; 2.1 Hz, 2H), 4.36 (*t*, *J*= 6; 9 Hz, 2H), 1.73-1.86 (*m*, 2H), 1.32-1.49 (*m*, 4H), 0.93 (*t*, *J*= 6; 9 Hz, 3H); **^{13}C NMR (100 MHz, $CDCl_3$): δ** 164.90, 150.60, 136.02, 130.80, 123.66, 66.25, 28.44, 28.25, 22.47, 14.10; **MS (ESI$^+$):** *m/z* calculated for $C_{12}H_{15}NO_4$: 237.252, found: 238.4 (M+H$^+$)

Heptyl 4-nitrobenzoate **(25)** Yield: 80%, **^{1}H NMR (300 MHz, $CDCl_3$): δ** 8.29 (*dt*, J_o= 9; J_m= 1.8; 2.1 Hz, 2H), 8.21 (*dt*, J_o= 9; J_m= 1.8; 2.1 Hz, 2H), 4.36 (*t*, *J*= 6 Hz, 2H), 1.73-1.84 (*m*, 2H), 1.24-1.50 (*m*,

8H), 0.89 (*t*, *J*= 6; 9 Hz, 3H); **^{13}C NMR (100 MHz, $CDCl_3$): δ** 164.90, 150.57, 136.00, 130.79, 123.66, 66.26, 28.72, 26.07, 29.05, 31.84, 22.72, 14.21; **MS (ESI$^+$):** *m/z* calculated for $C_{14}H_{19}NO_4$: 265.305, found: 266.5 (M+H$^+$)

Octyl 4-nitrobenzoate **(26)** Yield: 6%, **^{1}H NMR (300 MHz, $CDCl_3$): δ** 8.29 (*dt*, J_o= 9; J_m= 1.8; 2.1 Hz, 2H), 8.21 (*dt*, J_o= 9; J_m= 1.8, 2.1; 2.4 Hz, 2H), 4.37 (*t*, *J*= 6; 9 Hz, 2H), 1.23-1.50 (*m*, 2H), 1.23-1.50 (*m*, 10H), 0.89 (*t*, *J*= 6; 9 Hz, 3H), **^{13}C NMR (100 MHz, CDCl3): δ** 164.90, 150.65, 136.06, 130.80, 123.66, 66.27, 31.91, 28.75, 26.12, 29.35, 29.30, 22.77, 14.21; **MS (ESI$^+$):** *m/z* calculated for $C_{15}H_{21}NO_4$: 279.332, found: 280.3 (M+H$^+$)

Nonyl 4-nitrobenzoate **(27)** Yield: 26%, **^{1}H NMR (300 MHz, $CDCl_3$): δ** 8.29 (*dt*, J_o= 9; J_m= 1.8; 2.1; 2.4 Hz, 2H), 8.21 (*dt*, J_o= 9; J_m= 1.5; 1.8; 2.1 Hz, 2H), 4.37 (*t*, *J*= 6; 9 Hz, 2H), 1.73-1.86 (*m*, 2H), 1.21-1.51 (*m*, 12H), 0.88 (*t*, *J*= 6 Hz, 3H), **^{13}C NMR (100 MHz, $CDCl_3$): δ** 164.90, 150.65, 135.06, 130.80, 123.66, 66.27, 28.75, 26.12, 29.36 31.98, 29.60, 29.38, 22.79, 14.22; **MS (ESI$^+$):** *m/z* calculated for $C_{16}H_{23}NO_4$: 293.358, found: 294.4 (M+H$^+$)

1-Methyloctyl 4-nitrobenzoate (**28**) Yield: 93%, **^{1}H NMR (300 MHz, $CDCl_3$): δ** 8.30 (*d*, *J*= 12Hz, 2H), 8.21 (*d*, *J*= 12Hz, 2H), 5.20 (*m*, 1H), 1.77 (*m*, 1H), 1.65 (*m*, 1H), 1.38 (*d*, *J*=6Hz, 3H), 1.45 – 1.20 (*m*, 10H), 0.88 (*t*, *J*= 6Hz, 3H), **^{13}C NMR (100 MHz, $CDCl_3$): δ** 164.31, 150.13, 136.31, 130.62, 123.47, 73.16, 35.93, 31.76, 29.39, 29.17, 25.41, 22.62, 19.98, 15.9, 14.07; **MS (ESI$^+$):** *m/z* calculated for $C_{16}H_{23}NO_4$: 293.358, found: 294.6 (M+H$^+$)

Decyl 4-nitrobenzoate (**29**) Yield: 91%, **^{1}H NMR (300 MHz, $CDCl_3$): δ** 8.19 (*d*, *J*= 3Hz, 2H), 8.12 (*d*, *J*= 3Hz, 2H), 4.38 (*t*, *J*= 6Hz, 2H), 1.81 (*m*, 2H), 1.40 – 1.21 (*m*, 14H), 0.89 (*t*, *J*= 6Hz, 3H), **^{13}C NMR (100 MHz, $CDCl_3$): δ** 164.77, 150.47, 135.89, 130.67, 123.53, 66.14, 31.86, 29.53, 29.30, 29.25, 28.60, 25.98, 25.00, 22.68, 14.14, **MS (ESI$^+$):** *m/z* calculated for $C_{17}H_{25}NO_4$: 307.385, found: 308.3 (M+H$^+$)

Undecyl 4-nitrobenzoate (**30**) Yield: 78%, **^{1}H NMR (300 MHz, $CDCl_3$): δ** 8.30 (*d*, *J*= 3Hz, 2H), 8.22 (*d*, *J*= 3Hz, 2H), 4.29 (*t*, *J*= 6Hz, 2H), 1.72 (*m*, 2H), 1.46 – 1.21 (*m*, 16H), 0.80 (*t*, *J*= 6Hz, 3H), **^{13}C NMR (100 MHz, $CDCl_3$): δ** 164.75, 155.39, 143.82, 132.44, 128.78, 68.10, 31.93, 30.35, 29.58, 29.43, 29.17, 28.78, 27.64, 27.25, 22.69, 14.10, **MS (ESI$^+$):** *m/z* calculated for $C_{18}H_{27}NO_4$: 321.411, found: 322.6 (M+H$^+$)

Dodecyl 4-nitrobenzoate (**31**) Yield: 67%, **^{1}H NMR (300 MHz, $CDCl_3$): δ** 8.29 (*dt*, J_o= 9; J_m= 1.8; 2.1 Hz, 2H), 8.20 (*dt*, J_o= 9; J_m= 1.8; 2.1 Hz, 2H), 4.37 (*t*, *J*= 6; 9 Hz, 2H), 1.73-1.86 (*m*, 2H), 1.21-1.50 (*m*, 18H), 0.88 (*t*, *J*= 6; 9 Hz, 3H), **^{13}C NMR (100 MHz, $CDCl_3$): δ** 166.71, 155.79, 144.82, 132.99, 129.18, 68.70, 31.53, 30.15, 29.62, 29.50, 29.25, 28.90, 28.01, 27.14, 26.35, 22.68, 14.11, **MS (ESI$^+$):** *m/z* calculated for $C_{19}H_{29}NO_4$: 335.438, found: 336.4 (M+H$^+$)

Tridecyl 4-nitrobenzoate **(32)** Yield: 86%, **^{1}H NMR (300 MHz, $CDCl_3$): δ** 8.29 (*dt*, J_o= 9; J_m= 1.8; 2.1 Hz, 2H), 8.21 (*dt*, J_o= 9; J_m= 1.8; 2.1 Hz, 2H), 4.37 (*t*, *J*= 6 Hz, 2H), 1.73-1.85 (*m*, 2H), 1.22-1.50 (*m*, 20H), 0.88 (*t*, *J*= 6; 9 Hz, 3H). **^{13}C NMR (100 MHz, $CDCl_3$): δ** 164.90, 150.65, 136.06, 130.80, 123.65, 66.27, 28.75, 26.12, 29.38, 29.49, 32.06, 22.82, 14.24. **MS (ESI$^+$):** *m/z* calculated for $C_{20}H_{31}NO_4$: 349.464, found: 350.5 (M+H$^+$)

Tetradecyl 4-nitrobenzoate (**33**) Yield: 81%, **^{1}H NMR (300 MHz, $CDCl_3$): δ** 8.31 (*d*, *J*= 3Hz, 2H), 8.23 (*d*, *J*= 3Hz, 2H), 4.38 (*t*, *J*= 6Hz, 2H), 1.80 (*m*, 2H), 1.37 – 1.12 (*m*, 22H), 0.89 (*t*, *J*= 6Hz, 3H), **^{13}C NMR (100 MHz, $CDCl_3$): δ** 166.71, 155.79, 144.82, 132.99, 129.18, 68.70, 31.92, 29.65, 29.61, 29.57, 29.51, 9.35, 29.24, 28.91, 28.60, 25.98, 22.69, 14.12; **MS (ESI$^+$):** *m/z* calculated for $C_{21}H_{33}NO_4$: 363.491, found: 364.7 (M+H$^+$)

Butyl 3,5-dinitrobenzoate **(35)** Yield: 57%, **^{1}H NMR (300 MHz, $CDCl_3$): δ** 9.23 (*t*, *J*= 2.1 Hz, 1H), 9.15 (*d*, *J*= 3 Hz, 2H), 4.46 (*t*, *J*= 6 Hz, 2H), 1.77-1.88 (*m*, 2H), 1.43-1.55 (*m*, 2H), 1.01 (*t*, *J*= 6 Hz, 3H).**^{13}C NMR (100 MHz, $CDCl_3$): δ** 162.71, 148.64, 134.16, 129.54, 122.42, 66.99, 30.71, 19.30, 13.82. **MS (ESI$^+$):** *m/z* calculated for $C_{11}H_{12}N_2O_6$: 268.223, found: 269.3 (M+H$^+$)

Pentyl 3,5-dinitrobenzoate **(36)** Yield: 39%, **^{1}H NMR (300 MHz, CDCl$_3$): δ** 9.22 (*t*, J= 3 Hz, 1H), 9.16 (*d*, *J*= 3 Hz, 2H), 4.45 (*t*, *J*= 6; 9 Hz, 2H), 1.78-1.90 (*m*, 2H), 1.36-1.49 (*m*, 4H), 0.95 (*t*, *J*= 6 Hz, 3H).**^{13}C NMR (100 MHz, CDCl$_3$): δ** 162.71, 148.85, 134.35, 129.55, 122.43, 67.29, 28.39, 28.16, 22.44, 14.06. **MS (ESI$^+$):** *m/z* calculated for $C_{12}H_{14}N_2O_6$: 282.249, found: 283.4 (M+H$^+$)

Heptyl 3,5-dinitrobenzoate **(38)** Yield: 49%, **^{1}H NMR (300 MHz, CDCl$_3$): δ** 9.23 (*t*, *J*= 2.1 Hz, 1H), 9.16 (*d*, *J*= 3 Hz, 2H), 4.45 (*t*, *J*= 6 Hz, 2H), 1.78-1.89 (*m*, 2H), 1.28-1.49 (*m*, 8H), 0.90 (*t*, *J*= 6 Hz, 3H).**^{13}C NMR (100 MHz, CDCl$_3$): δ** 162.75, 148.84, 134.5, 129.45, 122.43, 67.30, 31.81, 29.02, 28.70, 26.00, 22.70, 14.17. **MS (ESI$^+$):** *m/z* calculated for $C_{14}H_{18}N_2O_6$: 310.303, found: 311.3 (M+H$^+$)

Octyl 3,5-dinitrobenzoate **(39)** Yield: 18%, **^{1}H NMR (300 MHz, CDCl$_3$): δ** 9.22 (*t*, *J*= 2.1 Hz, 1H), 9.16 (*d*, *J*= 3 Hz, 2H), 4.45 (*t*, *J*= 6 Hz, 2H), 1.78-1.89 (*m*, 2H), 1.25-1.50 (*m*, 10H), 0.89 (*t*, *J*= 6 Hz, 3H).**^{13}C NMR (100 MHz, CDCl$_3$): δ** 162.56, 148.69, 134.20, 129.39, 122.27, 67.15, 31.91, 29.16, 29.12, 28.54, 25.88, 22.61, 14.05. **MS (ESI$^+$):** *m/z* calculated for $C_{15}H_{20}N_2O_6$: 324.329, found: 325.3 (M+H$^+$)

Nonyl 3,5-dinitrobenzoate (**40**) Yield: 90%, **^{1}H NMR (300 MHz, CDCl$_3$): δ** 9.25 (*t*, *J*= 3Hz, 1H), 9.18 (*d*, *J* = 3Hz, 2H), 4.46 (*t*, *J*= 6Hz, 2H), 1.85 (*m*, 2H), 1.44 – 1.24 (*m*, 12H), 0.89 (*t*, *J*= 9Hz, 3H) **^{13}C NMR (100 MHz, CDCl$_3$): δ** 168.50, 147.18, 135.99, 129.11, 123.08, 67.77, 31.16, 29.21, 28.99, 28.47, 27.76, 25.89, 22.35, 14.14; **MS (ESI$^+$):** *m/z* calculated for $C_{16}H_{22}N_2O_6$: 338.356, found: 339.5 (M+H$^+$)

1-Methyloctyl 3,5-dinitrobenzoate (**41**) Yield: 88%, **^{1}H NMR (300 MHz, CDCl$_3$): δ** 9.24 (*t*, *J* = 3Hz, 1H), 9.17 (*d*, *J* = 3Hz, 2H), 5.26 (*m*, 1H), 1.79 (*m*, 1H), 1.68 (*m*, 1H), 1.36 (*d*, *J*= 3Hz, 3H), 1.41 – 1.28 (*m*, 10H), 0.88 (*t*, *J*= 6Hz, 3H), **^{13}C NMR (100 MHz, CDCl$_3$): δ** 161.10, 148.65, 134.60, 129.38, 122.18, 74.62, 35.83, 31.74, 29.34, 29.14, 25.44, 22.61, 19.97, 15.9, 14.06; **MS (ESI$^+$):** *m/z* calculated for $C_{16}H_{22}N_2O_6$: 338.356, found: 339.4 (M+H$^+$)

Decyl 3,5-dinitrobenzoate (**42**) Yield: 98%, **^{1}H NMR (300 MHz, CDCl$_3$): δ** 9.25 (*t*, *J*=3Hz, 1H), 9.18 (*d*, *J* = 3Hz, 2H), 4.46 (*t*, *J*= 6Hz, 2H), 1.85 (*m*, 2H), 1.49 – 1.22 (*m*, 14H), 0.89 (*t*, *J*= 9Hz, 3H), **^{13}C NMR (100 MHz, CDCl$_3$): δ** 162.56, 148.68, 134.19, 130.02, 122.28, 67.16, 31.86, 29.67, 29.53, 29.38, 29.25, 28.90, 28.50, 22.66, 14.09; **MS (ESI$^+$):** *m/z* calculated for $C_{17}H_{24}N_2O_6$: 352.382, found: 353.4 (M+H$^+$)

Undecyl 3,5-dinitrobenzoate (**43**) Yield: 72%, **^{1}H NMR (300 MHz, CDCl$_3$): δ** 9.24 (*t*, *J*=3Hz, 1H), 9.18 (*d*, *J* = 3Hz, 2H), 4.46 (*t*, *J*= 6Hz, 2H), 1.85 (*m*, 2H), 1.46 – 1.22 (*m*, 16H), 0.89 (*t*, *J*= 9Hz, 3H), **^{13}C NMR (100 MHz, CDCl$_3$): δ** 162.56, 148.68, 134.19, 129.41, 122.28, 67.16, 31.89, 29.58, 28.54, 28.15, 27.01, 26.70, 25.89, 22.67, 14.10 **MS (ESI$^+$):** *m/z* calculated for $C_{18}H_{26}N_2O_6$: 366.409, found: 367.5 (M+H$^+$)

Dodecyl 3,5-dinitrobenzoate (**44**) Yield: 85%, **^{1}H NMR (300 MHz, CDCl$_3$): δ** 9.15 (*t*, *J*= 3Hz, 1H), 9.09 (*d*, *J*= 3Hz, 2H), 4.38 (*t*, *J*= 6Hz, 2H), 1.85 (*m*, 2H), 1.49 – 1.12 (*m*, 18H), 0.89 (*t*, *J*= 9Hz, 3H), **^{13}C NMR (100 MHz, CDCl$_3$): δ** 165.94, 148.65, 134.22, 129.40, 122.99, 67.74, 38.89, 31.93, 29.65, 29.49, 29.37, 29.23 28.98, 22.97, 14.05 **MS (ESI$^+$):** *m/z* calculated for $C_{19}H_{28}N_2O_6$: 380.436, found: 381.4 (M+H$^+$)

Tridecyl 3,5-dinitrobenzoate (**45**) Yield: 55%, **^{1}H NMR (300 MHz, CDCl$_3$): δ** 9.22 (t, J= 3 Hz, 1H), 9.16 (d, J= 3 Hz, 2H), 4.45 (t, J= 6 Hz, 2H), 1.78-1.89 (m, 2H), 1.23-1.49 (m, 20H), 0.88 (t, J= 9 Hz, 3H), **^{13}C NMR (100 MHz, CDCl$_3$): δ** 162.71, 148.85, 134.36, 129.45, 122.42, 67.31, 28.70, 26.04, 29.36, 29.49, 32.06, 22.83, 14.25. **MS (ESI$^+$):** *m/z* calculated for $C_{20}H_{30}N_2O_6$: 394.462, found: 395.5 (M+H$^+$)

Tetradecyl 3,5-dinitrobenzoate (**46**) Yield: 78%, **^{1}H NMR (300 MHz, CDCl$_3$): δ** 9.16 (*t*, *J* = 3Hz, 1H), 9.09 (*d*.= 3Hz, 2H), 4.38 (*t*, *J*= 6Hz, 2H), 1.76 (*m*, 2H), 1.25 – 1.12 (*m*, 22H), 0.81 (*t*, *J*= 9Hz, 3H), **^{13}C NMR (100 MHz, CDCl$_3$): δ** 162.57, 148.66 134.17, 129.44, 122.31, 67.18, 31.93, 30.96,

30.57, 29.66, 29.63, 29.57, 29.49, 29.37, 29.23, 28.54, 25.90, 22.70, 14.14; **MS (ESI$^+$):** *m/z* calculated for $C_{20}H_{30}N_2O_6$: 408.489, found: 409.5 (M+H$^+$)

Hexadecyl 3,5-dinitrobenzoate **(47)** Yield: 28%, **^{1}H NMR (300 MHz, CDCl$_3$): δ** 9.24 (t, J = 2.1 Hz, 1H), 9.17 (d, J = 2.1 Hz, 2H), 4.47 (t, J = 6.8 Hz, 2H), 1.85 (m, 2H), 1.27 (m, 26H), 0.89 (t, J = 7.4, 3H). **^{13}C NMR (100 MHz, CDCl$_3$): δ** 162.66, 122.27, 134.18, 129.39, 148.74, 67.12, 31.96, 25.92, 14.10. **MS (ESI$^+$):** *m/z* calculated for $C_{20}H_{30}N_2O_6$: 436.542, found: 437.6 (M+H$^+$)

Butyl 3-nitro-5-(trifluoromethyl) benzoate **(48)** Yield: 84%, **^{1}H NMR (300 MHz, CDCl$_3$): δ** 9.05 (dd, J = 1.9; 2.0 Hz, 1H), 8.69 (dd, J = 2.3; 1.9 Hz, 1H), 8.63 (dd, J = 2.3; 2.0 Hz, 1H), 4.45 (t, J = 6.7 Hz, 2H), 1.91-1.75 (m, 2H), 1.60 – 1.34 (m, 2H) 0.98 (t, J = 7.4, 3H); **^{13}C NMR (100 MHz, CDCl$_3$): δ** 163.39, 148.62, 133.72, 132.03, 127.60, 124.52, 123.92, 121.21, 66.50, 30.59, 19.18, 13.84. **MS (ESI$^+$):** *m/z* calculated for $C_{12}H_{12}F_3NO_4$: 291.223, found: 292.6 (M+H$^+$)

Hexyl 3-nitro-5-(trifluoromethyl) benzoate **(49)** Yield: 71%, **^{1}H NMR (300 MHz, CDCl$_3$): δ** 9.05 (dd, J = 1.9; 2.1 Hz, 1H), 8.69 (dd, J = 2.1; 1.7 Hz, 1H), 8.63 (dd, J = 1.7; 2.0 Hz, 1H), 4.44 (t, J = 6.8 Hz, 2H), 1.84 (m, 2H), 1.55 – 1.25 (m, 6H) 0.93 (t, J = 7.4, 3H). **^{13}C NMR (100 MHz, CDCl$_3$): δ** 163.50, 120.61, 131.25, 127.57, 124.42, 148.84 ,133.85, 66.84, 31.38, 26, 14.05. **MS (ESI$^+$):** *m/z* calculated for $C_{14}H_{16}F_3NO_4$: 319.276, found: 320.5 (M+H$^+$)

Octyl 3-nitro-5-(trifluoromethyl) benzoate **(50)** Yield: 80%, **^{1}H NMR (300 MHz, CDCl$_3$): δ** 9.05 (dd, J = 1.9; 2.0 Hz, 1H), 8.69 (dd, J = 2.1; 2.0 Hz, 1H), 8.63 (dd, J = 1.7; 1.8 Hz, 1H), 4.44 (t, J = 6.8 Hz, 2H), 1.84 (m, 2H), 1.31 (m, 10H), 0.89 (t, J = 7.4, 3H). **^{13}C NMR (100 MHz, CDCl$_3$): δ** 163.20, 124.34, 133.29, 131.75, 127.71, 148.86 ,133.64, 66.76, 31.84, 25.91, 14.03. **MS (ESI$^+$):** *m/z* calculated for $C_{16}H_{20}F_3NO_4$: 347.329, found: 348.4 (M+H$^+$)

Decyl 3-nitro-5-(trifluoromethyl) benzoate **(51)** Yield: 75%, **^{1}H NMR (300 MHz, CDCl$_3$): δ** 9.05 (dd, J = 1.9; 2.0 Hz, 1H), 8.68 (dd, J = 2.2; 2.1 Hz, 1H), 8.63 (dd, J = 1.7; 1.8 Hz, 1H), 4.44 (t, J = 6.8 Hz, 2H), 1.91 – 1.76 (m, 2H), 1.36 – 1.24 (m, 10H), 0.95 – 0.82 (t, J = 7.4, 3H). **^{13}C NMR (100 MHz, CDCl$_3$): δ** 163.45, 148.74, 133.83, 133.19, 132.69, 127.45, 124.40, 66.82, 31.66, 25.91, 14.06. **MS (ESI$^+$):** *m/z* calculated for $C_{18}H_{24}F_3NO_4$: 375.383, found: 376.6 (M+H$^+$)

Dodecyl 3-nitro-5-(trifluoromethyl) benzoate **(52)** Yield: 78%, **^{1}H NMR (300 MHz, CDCl$_3$): δ** 9.05 (dd, J = 1.8; 1.9 Hz, 1H), 8.69 (dd, J = 2.0; 1.9 Hz, 1H), 8.63 (dd, J = 1.6; 1.8 Hz, 1H), 4.44 (t, J = 6.8 Hz, 2H), 1.91 – 1.76 (m, 2H), 1.29 (m, 20H), 0.95 – 0.84 (t, J = 7.4, 3H). **^{13}C NMR (100 MHz, CDCl$_3$): δ** 163.18, 120.70, 132.13, 127.35, 124.59, 148.77, 133.68, 67.02, 31.88, 25.93, 14.07. **MS (ESI$^+$):** *m/z* calculated for $C_{20}H_{28}F_3NO_4$: 403.436, found:404.6

S-Butyl Thiobenzoate **(53)** Yield: 46%; **^{1}H NMR (300 MHz, CDCl$_3$): δ** 8.04 – 7.94 (m, 2H), 7.64 – 7.51 (m, 1H), 7.51 – 7.39 (m, 2H), 3.10 (t, J = 7.3, 2H), 1.76 – 1.57 (m, 2H), 1.57 – 1.36 (m, 2H), 0.97 (t, J = 7.3 Hz, 3H). **^{13}C NMR (100 MHz, CDCl$_3$): δ** 192.23, 137.69, 134.16, 128.60, 127.05, 31.84, 28.73, 22.21, 12.88. **MS (ESI$^+$):** *m/z* calculated for $C_{11}H_{14}OS$: 194.294, found: 195.2 (M+H$^+$)

S-Octyl Thiobenzoate **(54)** Yield: 38%; **^{1}H NMR (300 MHz, CDCl$_3$): δ** 8.02 – 7.93 (m, 2H), 7.61 – 7.52 (m, 1H), 7.51 – 7.37 (m, 2H), 3.07 (t, J = 7.3, 2H), 1.75 – 1.60 (m, 2H), 1.49 – 1.21 (m, 10H), 0.89 (t, J = 6.7 Hz, 3H). **^{13}C NMR (100 MHz, CDCl$_3$): δ** 192.22, 137.28, 133.21, 128.56, 127.19, 31.82, 29.58, 29.15, 29.08, 28.97, 22.66, 14.12. **MS (ESI$^+$):** *m/z* calculated for $C_{15}H_{22}OS$: 250.400, found: 251.2 (M+H$^+$)

S-Dodecyl Thiobenzoate **(55)** Yield: 41%; **^{1}H NMR (300 MHz, CDCl$_3$): δ** 8.02 – 7.93 (m, 2H), 7.62 – 7.51 (m, 1H), 7.48 – 7.39 (m, 2H), 3.06 (t, J = 7.3, 2H), 1.77 – 1.59 (m, 2H), 1.51 – 1.18 (m, 18H), 0.88 (t, J = 6.7 Hz, 3H). **^{13}C NMR (100 MHz, CDCl$_3$): δ** 192.31, 137.40, 133.33, 128.68, 127.30, 32.06, 29.79, 29.73, 29.65, 29.49, 29.31, 29.20, 29.09, 22.83, 14.27. **MS (ESI$^+$):** *m/z* calculated for $C_{19}H_{30}OS$: 306.507, found: 307.6 (M+H$^+$)

S-Butyl 4-nitrothiobenzoate **(56)** Yield: 55%; **^{1}H NMR (300 MHz, CDCl$_3$): δ** 8.32 – 8.18 (ddd, J_o = 9 ; J_m = 1.8; 2.1 Hz, 2H, C3-H; C5-H), 8.10 – 7.99 (ddd, J_o = 9 ; J_m = 1.8; 2.1 Hz, 2H, C2-H; C6-H), 3.06 (t, J = 7.3

Hz, 2H), 1.70-1.4 (m, 2H), 1.49-1.29 (m, 2H), 0.90 (t, J = 7.3 Hz, 3H). **^{13}C NMR (100 MHz, CDCl$_3$): δ** 190.81, 150.71, 141.84, 128.18, 123.83, 31.50, 29.53, 22.02, 13.52. **MS (ESI$^+$):** *m/z* calculated for $C_{11}H_{13}NO_3S$: 239.292, found: 240.2 (M+H$^+$)

S-Octyl 4-nitrothiobenzoate **(57)** Yield: 28%; **^{1}H NMR (300 MHz, CDCl$_3$): δ** 8.37 – 8.26 (ddd, J_o = 9 ; J_m = 1.8; 2.1 Hz, 2H, C3-H; C5-H), 8.18 – 8.08 (ddd, J_o = 9 ; J_m = 1.8; 2.1 Hz, 2H, C2-H; C6-H), 3.14 (t, J = 7.3 Hz, 2H), 1.79-1.63 (m, 2H), 1.51-1.38 (m, 2H), 1.38 – 1.25 (m, 8H), 0.90 (t, J = 7.3 Hz, 3H). **^{13}C NMR (100 MHz, CDCl$_3$): δ** 190.82, 150.45, 141.98, 128.18, 123.83, 31.85, 29.92, 22.56, 14.08. **MS (ESI$^+$):** *m/z* calculated for $C_{15}H_{21}NO_3S$: 295.398, found: 296.4 (M+H$^+$)

S-Dodecyl 4-nitrothiobenzoate **(58)** Yield: 32%; **^{1}H NMR (300 MHz, CDCl$_3$): δ** 8.29 (d, J = 8.7 Hz, 2H, C3-H e C5-H), 8.11 (d, J = 7.3 Hz, 2H, C2-H e C6-H), 3.12 (t, J = 7.3 Hz, 2H), 1.77-1.62 (m, 2H), 1.50-1.18 (m, 18H), 0.90 (t, J = 6.9 Hz, 3H). **^{13}C NMR (100 MHz, CDCl$_3$): δ** 190.72, 150.58, 141.99, 128.33, 123.97, 32.05, 29.79, 29.70, 29.61, 29.43, 29.25, 29.04, 22.82, 14.25. **MS (ESI$^+$):** *m/z* calculated for $C_{19}H_{29}NO_3S$: 351.504, found: 352.3 (M+H$^+$)

S-Butyl 3,5-dinitrothiobenzoate **(59)** Yield: 75%; **^{1}H NMR (300 MHz, CDCl$_3$): δ** 9.24 (t, *J* = 2.1 Hz, 1H), 9.10 (d, *J* = 2.1 Hz, 2H), 3.23 (t, *J* = 7.3 Hz, 2H), 1.82 – 1.61 (m, 2H), 1.61 – 1.34 (m, 2H), 0.90 (t, J = 7.3 Hz, 3H). **^{13}C NMR (100 MHz, CDCl$_3$): δ** 188.12, 122.15, 140.12, 126.88, 148.88, 30.92, 30.02, 22.15, 13.56. **MS (ESI$^+$):** *m/z* calculated for $C_{11}H_{12}N_2O_5S$: 284.289, found: 285.2 (M+H$^+$)

S-Octyl 3,5-dinitrothiobenzoate **(60)** Yield: 44%; **^{1}H NMR (300 MHz, CDCl$_3$): δ** 9.24 (t, *J* = 2.1 Hz, 1H), 9.10 (d, *J* = 2.1 Hz, 2H), 3.22 (t, *J* = 7.3 Hz, 2H), 1.82 – 1.61 (m, 2H), 1.61 – 1.34 (m, 10H), 0.90 (t, J = 7.3 Hz, 3H). **^{13}C NMR (100 MHz, CDCl$_3$): δ** 188.34, 148.70, 140.10, 126.88, 122.15, 30.92, 30.02, 21.75, 14.11. **MS (ESI$^+$):** *m/z* calculated for $C_{15}H_{20}N_2O_5S$: 340.396, found: 341.6 (M+H$^+$)

S-Dodecyl 3,5-dinitrothiobenzoate **(61)** Yield: 36%; **^{1}H NMR (300 MHz, CDCl$_3$): δ** 9.22 (t, *J* = 2.1 Hz, 1H), 9.08 (d, *J* = 2.1 Hz, 2H), 3.20 (t, *J* = 7.3 Hz, 2H), 1.73 (p, *J* = 7.3 Hz, 2H), 1.51 – 1.19 (m, 18H), 0.88 (t, J = 7.3 Hz, 3H). **^{13}C NMR (100 MHz, CDCl$_3$): δ** 188.26, 148.85, 140.11, 127.04, 122.30, 32.05, 30.23, 29.77, 29.59, 29.49, 29.26, 29.22, 28.97, 22.83, 14.27. **MS (ESI$^+$):** *m/z* calculated for $C_{19}H_{28}N_2O_5S$: 396.502, found: 397.6 (M+H$^+$)

S-Butyl 3-nitro-5-(trifluoromethyl)thiobenzoate **(62)** Yield: 82%; **^{1}H NMR (300 MHz, CDCl$_3$): δ** 8.98 (m, 1H, C2-H), 8.69 (d, J = 2.02 Hz, 1H, C4-H), 8.53 (m, 1H, C6-H), 3.20 (t, J = 7.33 Hz, 2H), 1.80 – 1.65 (m, 2H), 1.59 – 1.42 (m, 2H), 0.88 (td, J = 1.81, 7.26 Hz, 3H). **^{13}C NMR (100 MHz, CDCl$_3$): δ** 189.08, 148.92, 134.02, 132.45, 127.78, 124.52, 121.75, 31.42, 30.19, 22.95, 14.16. **MS (ESI$^+$):** *m/z* calculated for $C_{12}H_{12}F_3NO_3S$: 307.290, found: 308.3 (M+H$^+$)

S-Octyl 3-nitro-5-(trifluoromethyl)thiobenzoate **(63)** Yield: 30%; **^{1}H NMR (300 MHz, CDCl$_3$): δ** 8.96 (t, J = 1.87 Hz, 1H, C2-H), 8.67 (t, J = 2.03 Hz, 1H, C4-H), 8.51 (d, J = 1.77 Hz, 1H, C6-H), 3.17 (t, J = 7.3 Hz, 2H), 1.80 – 1.62 (m, 2H), 1.50 – 1.20 (m, 10H), 0.88 (t, J = 6.71 Hz, 3H). **^{13}C NMR (100 MHz, CDCl$_3$): δ** 188.93, 148.88, 133.79, 132.62, 127.55, 124.57, 121.61, 31.81, 29.92, 29.74, 29.62, 29.37, 22.75, 14.09. **MS (ESI$^+$):** *m/z* calculated for $C_{16}H_{20}F_3NO_3S$: 363.396, found: 364.5 (M+H$^+$)

S-Dodecyl 3-nitro-5-(trifluoromethyl)thiobenzoate **(64)** Yield: 26%; **^{1}H NMR (300 MHz, CDCl$_3$): δ** 8.96 (t, J = 1.84 Hz, 1H, C2-H), 8.67 (t, J = 1.96 Hz, 1H, C4-H), 8.51 (br. s, 1H, C6-H), 3.17 (t, J = 7.31 Hz, 2H), 1.80 – 1.64 (m, 2H), 1.38 – 1.19 (m, 18H), 0.88 (t, J = 6.63 Hz, 3H). **^{13}C NMR (100 MHz, CDCl$_3$): δ** 189.21, 148.73, 133.48, 132.58, 126.93, 124.17, 121.17, 31.94, 29.94, 29.87, 29.81, 29.74, 29.66, 29.47, 29.13, 22.63, 14.19. **MS (ESI$^+$):** *m/z* calculated for $C_{20}H_{28}F_3NO_3S$: 419.502, found: 420.8 (M+H$^+$)
